# Children's Acquisition of Homogeneity in Plural Definite Descriptions

**DOI:** 10.3389/fpsyg.2019.02329

**Published:** 2019-11-06

**Authors:** Lyn Tieu, Manuel Križ, Emmanuel Chemla

**Affiliations:** ^1^Office of the Pro Vice-Chancellor (Research and Innovation), School of Education, MARCS Institute for Brain, Behaviour and Development, Western Sydney University, Penrith, NSW, Australia; ^2^Department of Linguistics, University of Vienna, Vienna, Austria; ^3^Laboratoire de Sciences Cognitives et Psycholinguistique, Département d'Etudes Cognitives, ENS, PSL University, EHESS, CNRS, Paris, France

**Keywords:** homogeneity, language acquisition, alternatives, scalar implicature, definite descriptions, quantification, plurals, maximality

## Abstract

Plural definite descriptions give rise to *homogeneity* effects: the positive *The trucks are blue* and the negative *The trucks aren't blue* are both neither true nor false when some of the trucks are blue and some are not, that is, when the group of trucks is not *homogeneous* with respect to the property of being blue (Löbner, 1987, 2000; Schwarzschild, 1994; Križ, 2015b). The only existing acquisition studies related to the phenomenon have examined children's comprehension only of the affirmative versions of such sentences, and moreover have yielded conflicting data; while one study reports that preschoolers interpret definite plurals *maximally* (Munn et al., 2006, see also Royle et al., 2018), two other studies report that preschoolers allow *non-maximal* interpretations of definite plurals where adults do not (Karmiloff-Smith, 1979; Caponigro et al., 2012). Moreover, there is no agreed upon developmental trajectory to adult homogeneity. In this paper, we turn to acquisition data to investigate the predictions of a recent analysis of homogeneity that treats homogeneous meanings as the result of a scalar implicature (Magri, 2014). We conducted two experiments targeting 4- and 5-year-old French-speaking children's interpretations of plural definite descriptions in positive and negative sentences, and tested the same children on standard cases of scalar implicature. The experiments revealed three distinct subgroups of children: those who interpreted the plural definite descriptions existentially and failed to compute implicatures; those who both accessed homogeneous interpretations and computed implicatures; and finally, a smaller subgroup of children who appeared to access homogeneous interpretations without computing implicatures. We discuss the implications of our findings, which appear to speak against the implicature theory as the adult-like means of generating homogeneous meanings.

## 1. Introduction

**Figure 1:**
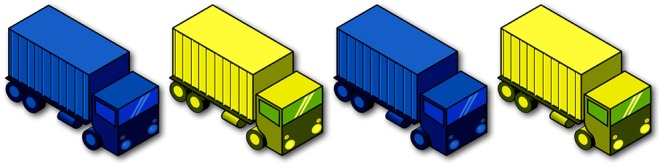
Image corresponding to a gap context. The first and third trucks are blue, while the second and fourth trucks are yellow.

Plural definite descriptions give rise to *homogeneity* effects (see among others, Fodor, 1970; Schwarzschild, 1994; Löbner, 2000; Breheny, 2005; Gajewski, 2005; Büring and Križ, 2013; Spector, 2013; Magri, 2014; Križ, 2015a). The positive (1) is true in a situation where all of the trucks are blue, but its negation (2) is only true in a situation where none of them are. There is a gap, however, in between these two possibilities; in a situation where some but not all of the trucks are blue (Figure 1), neither the positive sentence nor its negation are true. In this particular gap context, the group of trucks is not *homogeneous* with respect to the property of being blue^1^.

(1) The trucks are blue.
(2) The trucks are not blue.

Now compare (1) and (2) to the universally quantified (3) and (4). At first glance, the positive (1) might appear to be interpreted roughly equivalently to the universally quantified (3). Yet this apparent equivalency between *the*-NP and *all*-NP disappears under negation: in contrast to (2), the negative (4) is true in the scenario depicted in Figure 1.

(3) All of the trucks are blue.
(4) Not all of the trucks are blue.

The sentences with universal descriptions have complementary negations: the set of situations in which the positive sentence is true is the complement of the set of situations in which its negation is true, with no gap between them.

One way of conceptualizing the state of affairs for the definite descriptions is to say that in a gap scenario, both the positive and negative sentences are neither true nor false; rather they correspond to a third truth value, or to none at all. Some experimental evidence for this can be found in a study by Križ and Chemla (2015), who presented adults with such sentences as descriptions of situations that violated homogeneity. They reported that adults often assessed such descriptions as neither completely true nor completely false. In contrast, sentences containing universal descriptions like (3) did not display this gap, and were simply judged as completely false in the same non-homogeneous scenarios.

In the present paper, we investigate the acquisition of such *truth value gaps*. Building on Križ and Chemla (2015), we will take the pattern they observed in adults as the empirical hallmark of homogeneity: their adult participants assessed positive definite descriptions and their negations as non-true in gap contexts^2^. Now, if young children do not initially display this hallmark of homogeneity, one might expect them instead to assign complementary truth conditions to the positive and negative counterparts. In particular, one might expect children to liken plural definite descriptions (5) to existential quantifiers (6) or to universal quantifiers (7).

(5) a. The trucks are blue.
b. The trucks aren't blue.

(6) a. There are some blue trucks.
b. There aren't any blue trucks.

(7) a. Every truck is blue.
b. Not every truck is blue.

**Figure 2:**
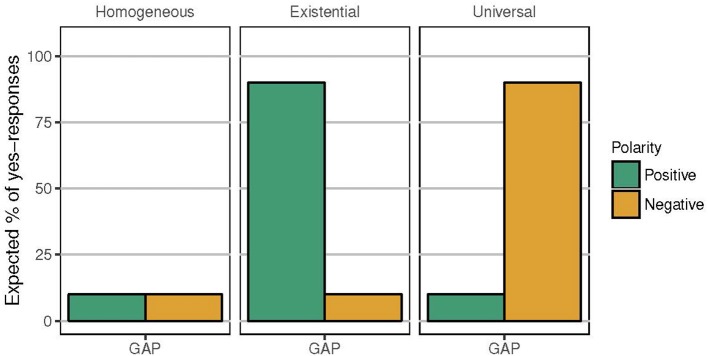
Expected response patterns for positive and negative definite descriptions such as *The trucks are blue* and *The trucks aren't blue* in gap contexts such as Figure 1, according to the interpretation of the plural definite description.

A child who is presented with (5) in a gap context like Figure 1, then, might be expected to respond in one of three ways, depending on the interpretation assigned to the plural definite. First, if the child is adult-like, she can be expected to treat the positive and negative descriptions uniformly, likely rejecting both as descriptions of gap contexts. This possibility corresponds to the homogeneous pattern depicted in Figure 2. Second, the child could interpret the definite existentially, i.e., in parallel with (6), prompting her to accept the positive sentence but not the negative sentence as a good description of Figure 1. This corresponds to the existential pattern depicted in Figure 2. Third, the child could interpret the definite universally, in line with (7), prompting her to accept the negative but not the positive description, as in the universal pattern in Figure 2^3^.

To our knowledge, there are only three existing studies that have specifically investigated children's comprehension of plural definite descriptions, examining in particular whether children assign *maximal* interpretations to plural definite descriptions. Karmiloff-Smith (1979) and Caponigro et al. (2012) report corroborating findings that children allow *non-maximal* interpretations of positive plural definite descriptions where adults would not. Such children would be expected to accept a sentence like (1) as a description of a context like Figure 1. While these previous experiments leave open the status of negative descriptions like (2), a problem that we will address shortly, they nevertheless provide a preliminary suggestion that children may not be sensitive to the truth value gap described above.

Karmiloff-Smith (1979) and Caponigro et al. (2012) provide different characterizations of their child participants' failure to enforce maximality in their interpretations of plural definite descriptions, although neither characterization provides an explanation of how children acquire maximality, nor of why it emerges relatively late (reportedly after 6 years of age). To date, there exists no unified explanation for these previous findings. Since these two studies were conducted, however, a recent semantic analysis of homogeneity has emerged which invokes a connection between the homogeneity that is triggered by plural definite descriptions and the enrichment mechanism that underlies the derivation of scalar implicatures (Magri, 2014). In what follows, we will investigate the precise predictions that such an analysis makes for children's development both of homogeneity and scalar implicatures, through two novel experiments.

The remainder of this paper is organized as follows. We will begin by briefly outlining the existing analyses of homogeneity in the semantics literature. We will then review the existing acquisition studies of plural definite descriptions, which raise as-of-yet unanswered questions about children's early interpretations of plural definite descriptions and about the learnability of homogeneity more generally. Since the scalar implicature account makes concrete predictions that one can test, we proceed to present two experiments where we did just that. We then discuss the implications of our findings for a theory of homogeneity and for the developmental trajectory toward adult homogeneity^4^.

## 2. Theories of Homogeneity

A few accounts of homogeneity have been proposed in the formal semantics literature. The earliest proposals treat homogeneity as a presupposition (Schwarzschild, 1994; Löbner, 2000; Gajewski, 2005). The general idea is that sentences like (1) and (2) carry a presupposition that either all of the trucks are blue or none of the trucks are blue. Since this presupposition is not satisfied in gap contexts like Figure 1, such descriptions give rise to a truth value gap.

A second approach is to say that there is some sort of indeterminacy or vagueness about the interpretation of the definite description, which itself might be either existential or universal. A sentence is then perceived as having a definite truth value if it has the same truth value no matter how this indeterminacy is resolved (Spector, 2013; Križ and Spector, 2017). For example, if *the trucks* in (1) can be interpreted either existentially or universally, we have two possible interpretations for the sentence:

(8) a. Some of the trucks are blue.
b. All of the trucks are blue.

The sentence in (1) is then true if both (8a) and (8b) are true, i.e., if all of the trucks are blue, and false if both (8a) and (8b) are false, i.e., if none of the trucks are blue. In Figure 1, neither condition is satisfied, and so (1) can be neither true nor false. The same reasoning applies to the negative sentence (2), since the negations of (8a) and (8b) are neither both true nor both false.

A third approach derives homogeneity as a scalar implicature. Magri (2014) proposes that plural definites have a literal existential meaning that is strengthened to the universal meaning through an implicature^5^. Take the example of the scalar implicature in (9).

(9) a. Some of the trucks are blue.
b. ⇝ *Not all of the trucks are blue*

The implicature in (9b) arises as the consequence of comparing the assertion in (9a) with alternatives that could have been uttered but were not. Assuming speakers are as informative as they can be (Grice, 1975), the speaker's choice to utter (9a), as opposed to the stronger alternative *All of the trucks are blue*, can lead us to conclude that this stronger alternative is false, generating the scalar implicature in (9b). This process by which the scalar implicature is derived can be analyzed as involving a covert exhaustification operator exh, roughly equivalent to a silent “only” (Fox, 2007; Chierchia et al., 2011):

(10) exh(Some of the trucks are blue) = Some of the trucks are blue and not all of the trucks are blue

According to Magri (2014), homogeneity can be derived by recursively applying this exhaustification procedure. Assume first that the definite plural *the trucks* has a plain existential meaning, much like *some trucks* in (9a). Assume further that the lexical alternatives for the definite include “some” (though crucially not “all”). Now if we apply the same exhaustification procedure as in (10), but do so recursively, we effectively arrive at a universal meaning for (1), as in (11).

(11) exh(exh(The trucks are blue)) = exh(The trucks are blue) and not(exh(some of the trucks are blue)) = Some of the trucks are blue and not(some but not all of the trucks are blue) = All of the trucks are blue

Of the three accounts outlined above, the scalar implicature account of homogeneity is of particular interest from a developmental perspective, in part because there exists a considerable amount of previous literature on the acquisition of scalar implicatures. This previous work will afford us a convenient means to empirically compare the two phenomena in development, and in doing so, to test the predictions of the theory^6^.

### 2.1. Testing the Predictions of the Implicature Account

An implicature account of homogeneity *prima facie* predicts that children should perform on homogeneity the way that they perform on implicatures. After all, the same mechanism would underlie the strengthened meaning of a scalar term like “some” and the strengthened homogeneous meaning of a plural definite description.

There have been a number of developmental studies focusing on implicatures. Many of the existing studies have reported that children typically compute fewer scalar implicatures than adults (see among many others, Braine and Rumain, 1981; Chierchia et al., 2001; Gualmini et al., 2001; Noveck, 2001; Papafragou and Musolino, 2003; Barner et al., 2011). More recent developmental work on implicatures has shown that children's success on implicatures can in fact vary considerably, depending on factors such as the methodology being used to test the child's knowledge of implicature, the particular scale being tested, and the kinds of experimental contexts in which the scalar items are presented. For example, Katsos and Bishop (2011) have shown that providing 5-year-old children with three graded response options vastly improves the children's performance on implicatures, compared to when they are presented with the more traditional binary yes/no response options. That is, when children are given the option to reward a puppet with a minimal, intermediate, or maximal reward, they tend to perform in more of an adult-like manner, offering the intermediate reward for literally true but underinformative statements. Katsos and Bishop (2011) propose that children are simply more *pragmatically tolerant* than adults are when forced to decide whether or not to accept an underinformative statement.

Another proposal that has gained traction in the developmental literature is the idea that children's performance on implicatures is somehow linked to the nature of the *alternatives* that are involved in computing the implicature, with potential difficulties arising from accessing lexical alternatives or understanding their relevance in a given context (Barner et al., 2011; Singh et al., 2016; Skordos and Papafragou, 2016; Tieu et al., 2016). In particular, children appear to exhibit greater difficulties with implicatures that involve *lexical replacement* of alternative scalar terms, e.g., *some*/*all, or*/*and*, and *might*/*must*. By contrast, children have been reported to successfully compute *ad hoc* implicatures (“My friend has glasses” ⇝ *My friend doesn't have both glasses and a hat*) (Stiller et al., 2015) and free choice inferences (“Kungfu Panda may push the green car or the red car” ⇝ *Kungfu Panda may push the green car and Kungfu Panda may push the red car*) (Tieu et al., 2016), as well as conjunctive inferences from disjunction (“The chicken pushed a bus or an airplane” ⇝ *The chicken pushed a bus and an airplane*) (Singh et al., 2016; Tieu et al., 2017). These inferences share a common property: they do not involve lexical replacement; rather, children can retrieve the required alternatives directly from the test sentences or from the experimental context.

Given the insights of these recent studies on implicatures, we will set out to test the implicature account of homogeneity in a carefully controlled, systematic way, keeping in mind the role that alternatives, methodology, and context can play. We will systematically compare homogeneity to an implicature that, on the implicature theory, actually corresponds to a *sub-computation* of the homogeneity implicature [recall that (10) is a sub-computation of (11)]. Importantly, we will also use exactly the same tasks and contexts to test the two phenomena. This means that whatever effect the context may have on the one, it should also have the same effect on the other. Moreover, because the lexical alternatives involved in generating the *not-all* implicature and the homogeneity implicature are the very same, i.e., “some” and “all”, we do not have to worry that children may acquire the alternatives for one inference earlier than for the other. In fact, the implicature theory in this case makes very straightforward, testable predictions.

If homogeneity is derived using the same mechanism as classical scalar implicatures, one should expect children to display sensitivity to homogeneity only once they are able to compute scalar implicatures, and more specifically only once they are able to compute the *not-all* implicature, since this corresponds to a sub-computation of the implicature of homogeneity. Previous studies have shown that without special training or facilitation, preschoolers typically respond to “some” statements in a manner consistent with the literal existential interpretation of the quantifier (e.g., Papafragou and Musolino, 2003). The implicature theory therefore predicts a similar pattern for homogeneity for such children, namely literal, existential interpretations of plural definite descriptions. Only once the children are capable of computing the *not-all* implicature will they display homogeneity effects. Furthermore, the implicature that gives rise to homogeneity effects involves *recursive* application of the exhaustification operator, so one might expect to see homogeneity surface even later in development than the regular first-order *not-all* implicature. While there is independent evidence that children are capable of recursive exhaustification (Zhou et al., 2013; Tieu et al., 2016), crucially, the timing prediction remains the same: we should not observe homogeneity surfacing *before* the scalar implicature.

## 3. Acquisition of Homogeneity

While there are existing studies of the acquisition of definite noun phrases on the one hand (see among others, Maratsos, 1974, 1976; Schafer and de Villiers, 2000; Matthewson et al., 2001; Pérez-Leroux et al., 2004; Schaeffer and Matthewson, 2005; Schmerse et al., 2014), and of plurality on the other hand (e.g., Berko, 1958; Winitz et al., 1981; Mervis and Johnson, 1991; Marcus et al., 1992; Fenson et al., 1994; Marchman et al., 1997; Sauerland et al., 2005; Barner et al., 2007; Zapf and Smith, 2008; Wood et al., 2009; Tieu et al., 2014; Davies et al., 2016), few studies have examined the two phenomena in conjunction. To our knowledge, there are only three existing studies that have specifically investigated children's comprehension of plural definite descriptions.

### 3.1. Karmiloff-Smith (1979)

In the earliest of these studies, Karmiloff-Smith (1979) reports a series of experiments investigating French-speaking children's production and comprehension of different kinds of noun phrases, including definite plural noun phrases. Two of Karmiloff-Smith's studies are relevant for our purposes here. First, she conducted a production study in which a child was prompted to produce directives, such as *Il faut mettre les camions dans le garage* “One must put the trucks in the garage.” The experimental set-up involved two experimenters. One experimenter (E2) would turn his back and close his eyes. The other experimenter (E1) would manipulate a series of objects, for example, moving a set of toy trucks into the garage. The child would then have to tell E2 what he would have to do to replicate that action. E1 would return the objects to their original locations, and E2 would then turn around and open his eyes, and carry out the action based on the child's directive. By manipulating what sets of objects were moved into the garage, the experimenters aimed to elicit different kinds of noun phrases from the child, e.g., *les camions* “the trucks,” *mes camions* “my trucks,” *les camions bleus* “the blue trucks,” etc. The experimenters tested children between the ages of 4;07 and 11;05. Karmiloff-Smith reports that for 4- and 5-year-olds, the definite article *les* was used to mark pluralization but not “totalization”; that is, *les X* was taken to signify any plural amount of X's, though not necessarily all the X's.

In a comprehension experiment modeled similarly to the production experiment, children were on the receiving end of the directives, and had to manipulate toy objects in response to these directives. For instance, they would hear sentences such as *Mets les voitures au garage* “Put the cars in the garage.” Karmiloff-Smith reports low percentages of correct responses from 4- and 5-year-olds, suggesting again that the definite *les X* for these children signified any plurality of X's, though not necessarily the full set of X's. More generally, Karmiloff-Smith proposes that children initially mark newly acquired functions, such as pluralization, or totalization, through separate morphemes. In the earliest stage, between 3 and 5.5 years of age, children associate the plural definite *les* only with pluralization. In a second stage, between 5 and 8 years of age, children add the universal marker *tous* ‘all' to convey totalization. Finally, after the age of 8 years, the definite plural *les* comes to simultaneously convey pluralization and totalization.

While Karmiloff-Smith's (1979) data are suggestive of what we have referred to in section 1 as the existential pattern of interpretation, notice that the experiments she reports did not include plural definite descriptions under negation. The study therefore leaves open the status of children's interpretation of the negations of such plural definite descriptions, and does not allow us to fully determine which of the three scenarios in Figure 2 the child's initial state corresponds to.

### 3.2. Munn et al. (2006)

The second study that has examined children's understanding of plural definite descriptions is reported in Munn et al. (2006). These authors compared children's understanding of singular and plural definite descriptions and indefinite nominals in English and Spanish. Like Karmiloff-Smith's comprehension study, Munn et al.'s study employed an act-out task. Preschoolers (mean age 4;01) were issued requests, such as “give me *the frogs next to the barn*”, where there was a set of toy frogs beside a toy barn. The authors report that almost all children gave the maximal element of the relevant set of frogs 95% of the time^7^. In contrast to the conclusions reached in Karmiloff-Smith (1979), the authors conclude that children correctly interpret plural definite descriptions maximally by the age of 3 years.

As pointed out in later work by Caponigro et al. (2010, 2012), however, there are some issues with this study. First, there were no control trials involving descriptions such as *some of the frogs*, so it is not clear whether children would also select the maximal set for such requests. Second, Caponigro et al. (2010) point out that Munn et al.'s reported percentage of maximal responses was calculated after excluding children who gave only one item in response to the plural definite description request; these children were clearly not assigning a maximal interpretation to the plural definite description. Third, Caponigro et al. (2012) point out that since Munn et al. (2006) did not provide a breakdown of the data by age, it is difficult to draw conclusions about when maximality in plural definite descriptions is acquired. Finally, like Karmiloff-Smith's (1979) study, this study, too, leaves open the status of children's interpretation of the negative definite description counterparts, without which we cannot tell whether the reported “maximal” behavior is due to an adult (homogeneous) interpretation of the plural definite description, or merely to a universal interpretation of the definite description.

### 3.3. Caponigro et al. (2012)

Caponigro et al. (2010, 2012) set out to investigate the possible developmental connection between plural definite descriptions like *the things on the plate* and free relative clauses like *what is on the plate*. The authors first conducted a Truth Value Judgment Task (TVJT) (Crain and Thornton, 1998) with 4-, 5-, 6-, and 7-year-old children, and a group of adult controls. In this task, participants were introduced to a character (Cookie Monster) who loves cookies but strongly dislikes onions. On critical target trials, children were presented with a picture of a plate containing three cookies and three onions, and were asked questions such as “Does Cookie Monster like *the things on the plate*?” or “Does Cookie Monster like *what's on the plate*?” The authors report that overall, free relatives and plural definite descriptions were interpreted maximally more frequently than existential nominals containing *one* and *some*, but less frequently than those containing the universal *all*^8^. As the authors point out, there are a couple of reasons to pursue the investigation further. First, even the adult controls that they tested did not always access maximal readings for the plural definite descriptions and free relatives, making it difficult to assess children's performance on the task. The authors suggest the problem may lie in the nature of the TVJT; they reason that if the plural definite descriptions introduced a presupposition of homogeneity, this presupposition was necessarily violated on the critical “mixed plate” trials, and so there could be no true or false answer given to the critical test questions. A second issue that the authors point out is that up until age 7, participants' responses to the critical trials were not different from chance; it is therefore unclear whether participants were simply guessing at random. Finally, as we pointed out previously for Munn et al.'s study, a maximal answer to positive sentences could be obtained either through homogeneity, or through a mere universal (non-adult-like) interpretation of the plural definite description.

To address the potential felicity issue with the use of the TVJT, the authors next conducted an act-out task, again with 4-, 5-, 6-, and 7-year-olds, and adult controls. In this task, participants were issued requests such as “Can you give me *the things on the plate*?” and “Can you give me *what's on the plate*?” The authors also compared the target conditions with ones in which the request contained *some, all*, and the nonsense determiner *blick*. Two of their main findings are relevant for us here. First, the authors reported a significant main effect of Question Type, with plural definite description responses differing from those in the *some* and *all* conditions. Second, further analysis revealed that the responses of the 4- and 5-year-olds, but not those of the 6- and 7-year-olds, were significantly different from those of adults; crucially, 4- and 5-year-olds assigned fewer maximal interpretations to the plural definite descriptions than the older children or the adults.

Caponigro et al. propose that although young children are capable of representing plural individuals, they struggle to map the conceptual/semantic representations of plural individuals to the relevant linguistic structure. These authors assume that the definite determiner denotes a function that applies to a set of individuals and returns the maximal element of that set (Link, 1983). They propose that young children associate the plural noun phrase with a set containing a plurality of atomic individuals, but one that contains no plural individuals or maximal individual. The meaning of *the* cannot apply to a set lacking a maximal individual, and so the semantic derivation fails, leading to the absence of maximal interpretations. The authors suggest that 4-year-olds must adopt other (possibly non-grammatical) strategies to deal with this failure, and point to the fact that their 4-year-olds treated the plural definite descriptions the same as they did the nonsense determiner *blick*.

### 3.4. Taking Stock

The previous acquisition studies described above tackled the question of whether young children enforce maximal interpretations on plural definite descriptions. The findings of Karmiloff-Smith (1979) and Caponigro et al. (2012) align, revealing that both French- and English-speaking children fail to interpret plural definite descriptions maximally until at least 6 years of age.

The findings of both of these studies raise three important questions. First, what underlies young children's non-maximal interpretations of plural definite descriptions? Second, what is the developmental trajectory that children take toward maximal interpretations? Finally, what triggers maximal interpretations, and so late in development? The two studies that report non-maximal behavior do not readily provide an answer to the third question, nor do they agree on the answers to the first two questions. On Karmiloff-Smith's proposal, children in the earliest stages associate the plural definite description with plurality, and not maximality. Children subsequently develop knowledge of the totalization function, and only later allow the plurifunctional/simultaneous marking of pluralization and totalization through the same morpheme. On Caponigro et al.'s proposal, children initially fail to access maximal interpretations because they associate the plural noun phrase with a set of plural atomic individuals that lacks a maximal individual. What is missing, the authors speculate, is an adult-like mapping between the target linguistic structure and the relevant conceptual representation.

The finding that young children as a group do not interpret plural definite descriptions as maximally as adults do, does not rule out the possibility that they nevertheless interpret these expressions in systematic ways, and in particular, in a manner consistent with one of the possibilities presented in Figure 2. Unfortunately, none of the previous studies allow us to determine which scenario in Figure 2 young children fall into, since these studies did not examine plural definite descriptions under negation^9^. Moreover, the data from these previous studies hint at more than one possibility. Specifically, Karmiloff-Smith's and Caponigro et al.'s participants who gave non-maximal responses could conceivably have assigned an existential interpretation to the plural definite description; Munn et al.'s participants, who gave maximal responses, could have interpreted the definite plural either universally or homogeneously. The first goal of our study, then, is to resolve this uncertainty surrounding the interpretations children assign to plural definite descriptions. In order to do so, we will examine children's interpretation of plural definite descriptions in both positive (upward-entailing) and negative (downward-entailing) declarative sentences. By examining *individual* participants' *pairs of responses* to both positive and negative plural definite descriptions in gap contexts, we will be able to identify whether they are assigning a homogeneous, existential, or universal interpretation to the plural definite descriptions.

The second main goal of the study is to pursue a characterization of the developmental trajectory to adult homogeneity, by investigating a potential connection with scalar implicatures. We will test Magri's (2014) scalar implicature theory of homogeneity through acquisition, by directly comparing individual children's performance on the two phenomena, using minimally different stimuli. In particular, we will investigate the timeline predictions that the account makes, specifically that we may observe the concurrent emergence of homogeneity and the *some-but-not-all* scalar implicature, or the emergence of the scalar implicature before homogeneity, but crucially not the emergence of homogeneity before the scalar implicature^10^.

A final difference we should point out between the previous studies and the present one concerns the tasks presented to the children. The production, act-out, and truth value judgment tasks used in Karmiloff-Smith (1979), Munn et al. (2006), and Caponigro et al. (2012) all involved some degree of reasoning about someone else's desires and actions. On the act-out tasks, children had to satisfy the demands of an issued request; they therefore had to decide how much action they would have to take in order to satisfy the speaker's desires. On the production task, children had to decide how much information to give in order for a third party to successfully carry out an action the way a second party had modeled it. On the TVJT, children had to assess the depicted scenarios against Cookie Monster's likes and dislikes. We make no claims about how adept children are at this kind of reasoning; we will, however, attempt to avoid this extra step entirely, and simplify the task by asking children to judge very simple descriptions of pictures of familiar objects.

## 4. Experiment 1

We designed a Truth Value Judgment Task to assess the interpretations that adults and children assign to positive and negative sentences containing plural definite descriptions. Participants' responses to the positive and negative descriptions in gap contexts would allow us to determine whether they interpreted the definite plural homogeneously, existentially, or universally. To investigate the predictions of the scalar implicature account of homogeneity (Magri, 2014), we also tested participants' interpretation of *some*-sentences in contexts that made the *not-all* implicature false. The direct comparison between homogeneity and scalar implicatures would allow us to assess the potential developmental connection between homogeneity and scalar implicatures.

### 4.1. Methods

Ethical approval for this study was obtained from the CERES (“Comité d'évaluation éthique des projets de recherche en santé non soumis à CPP”) under approval number 2013/46. Written informed consent was obtained from the parents or guardians of all child participants; adult participants were tested through an anonymous web-based survey, and had to click a button to provide informed consent before starting the experiment.

#### 4.1.1. Participants

We tested 24 French-speaking children (13 female) (4;04, 15−5;03, 24, *M* = 4;09) at two preschools in Paris. Two additional children were excluded because they answered fewer than six of eight control trials correctly (trials in which a sentence with a definite description was made uncontroversially true or uncontroversially false). The inclusion criterion of 75% accuracy on controls is fairly standard in truth value judgment task experiments of this kind, and was decided upon prior to testing. We also tested 22 adult native speakers of French, recruited through the online platform FouleFactory, at a total cost of € 57.60. All adults passed the controls and were included in the analysis.

#### 4.1.2. Procedure

Children were introduced to a puppet named Raffie the Giraffe, who interacted via webcam. Children were told that Raffie was still very little, and not very good at paying attention. They were then presented with a series of pictures, each containing four objects, and were asked to identify the colors of each of the four objects. The puppet was then asked to say something about the objects, and would utter a test sentence containing a plural definite noun phrase (e.g., *les ballons* “the balloons”), an existentially quantified noun phrase (e.g., *certains ballons* “some balloons”), or a universally quantified noun phrase (e.g., *tous les ballons* “all of the balloons”). Children had to judge the puppet's description and indicate their judgment by stamping on a score sheet, either under a happy face or a sad face.

Children were tested individually away from their classrooms. Responses were videorecorded for subsequent analysis. Children saw two training items involving the description of single, colored objects (i.e., a pink chair and a green piano), followed by 24 test trials presented in one of two pseudorandomized orders, one the reverse of the other (the order of presentation was counterbalanced across participants). The total task took roughly 10 min for children to complete.

Adults were tested on a web-based version of the task; the procedure and the visual stimuli were the same, but the sentences were presented visually (in the form of speech bubbles beside the puppet's picture) rather than orally. Adult participants indicated their responses by clicking on appropriate yes/no buttons.

#### 4.1.3. Materials

As we will describe in more detail below, participants received two training items, six homogeneity targets, eight uncontroversially true/false plural definite description controls, six universal quantification controls, and four scalar implicature targets. The full set of test sentences is provided in the Appendix.

**Figure 3:**
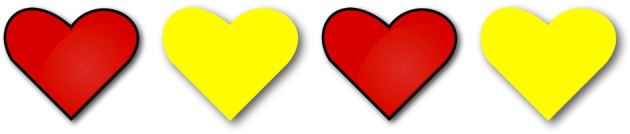
Example of an image presented in the gap condition. The first and third hearts are red, while the second and fourth hearts are yellow. The corresponding homogeneity target sentence was either *Les coeurs sont rouges* “The hearts are red” or *Les coeurs ne sont pas rouges* “The hearts are not red”.

***Homogeneity targets***. Participants heard three positive and three negative *les* “the”-NP sentences such as (12), presented in gap contexts such as Figure 3, in which only two of the four objects in the image were of the color indicated in the test sentence^11^.

(12) a. Les coeurs sont rouges.
  “The hearts are red.”
b. Les coeurs ne sont pas rouges.
  “The hearts are not red.”

If children treated the plural definite description as imposing homogeneity, they were expected to reject both the positive and the negative *the*-sentence, in accordance with the homogeneous pattern in Figure 2. If children interpreted the definite plural existentially, they were expected to accept the positive *the*-sentence but to reject the negative *the*-sentence. In contrast, if they interpreted it universally, they were expected to reject the positive but to accept the negative sentence. Participants saw three repetitions of the positive definite descriptions and three repetitions of the negative.

**Figure 4:**
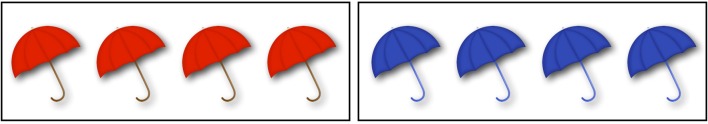
Images corresponding to the plural definite description control condition. When accompanied by the image on the left, in which all four umbrellas are red, the positive and negative descriptions in (13) would be associated with a *yes*- and a *no*-target, respectively. When accompanied by the image on the right, in which all four umbrellas are blue, the positive and negative sentences in (13) would be associated with a *no*- and a *yes*-target, respectively.

***Homogeneity controls***. In addition to the six homogeneity targets, participants also heard four positive and four negative definite descriptions like (13), presented in contexts that satisfied homogeneity (Figure 4); these allowed us to ensure that children understood basic plural definite descriptions, and in particular, could provide *yes*- and *no*-responses appropriately when there were no issues of non-homogeneity. In all contexts, where all of the objects shared the same color, the positive control was associated with a *yes*-target, and the negative with a *no*-target. In none contexts, where none of the objects had the color indicated in the test sentence, the positive definite description was associated with a *no*-target, and the negative with a *yes*-target.

(13) a. Les parapluies sont rouges.
  “The umbrellas are red.”
b. Les parapluies ne sont pas rouges.
  “The umbrellas are not red.”

The targets for these definite control trials were selected dynamically based on children's responses to the target sentences. Every third trial corresponded to a dynamic control, for which the experimenter could select either the *yes*- or the *no*-target. This precaution allowed us to ensure that participants could give both *yes*- and *no*-responses where appropriate, and allowed us to avoid overly long sequences of successive *yes*- and *no*-targets, which otherwise might encourage a *yes*- or *no*-bias, respectively (for previous examples of the use of such dynamic fillers, see Musolino and Lidz, 2006; Conroy et al., 2009; Tieu and Lidz, 2016; Lewis et al., 2017). Any participant who failed to correctly answer at least six of the eight definite plural controls was excluded from analysis.

Finally, we included a universal quantification condition, which contained three positive and three negative universally quantified descriptions such as (14), presented in gap contexts such as Figure 3. These would allow us to ensure that children could assign an adult-like, negation-preserving meaning to universally quantified sentences, and would provide a point of comparison for the plural definite descriptions.

(14) a. Tous les coeurs sont rouges.
  “All the hearts are red.”
b. Pas tous les coeurs sont rouges.
  “Not all the hearts are red.”^12^

**Figure 5:**
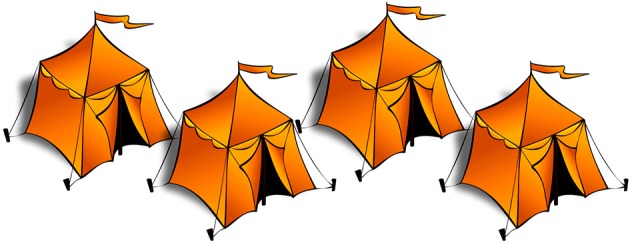
Example of an image presented in the scalar implicature target condition. All four of the tents are orange, while the corresponding test sentence was *Certaines tentes sont oranges* “Some tents are orange.”

***Scalar implicature targets***. To assess Magri's (2014) scalar implicature-based account of homogeneity, we also administered a scalar implicature test. Participants received four scalar implicature trials, which involved existentially quantified *certains* “some”-sentences, presented in contexts where all four objects displayed were of the mentioned color (Figure 5). If participants computed the *some-but-not-all* implicature, they were expected to reject the test sentences. If they accessed only the literal plain existential meaning of the sentences, however, they were expected to accept the descriptions. This condition would allow us to directly compare participants' performance on homogeneity and scalar implicatures.

***Summary of the materials***. In all, participants received two training items, six homogeneity targets, eight uncontroversially true/false plural definite description controls, six universal quantification controls, and four scalar implicature targets. The full set of test sentences is provided in the Appendix.

### 4.2. Results

#### 4.2.1. Plural Definite Descriptions

**Figure 6:**
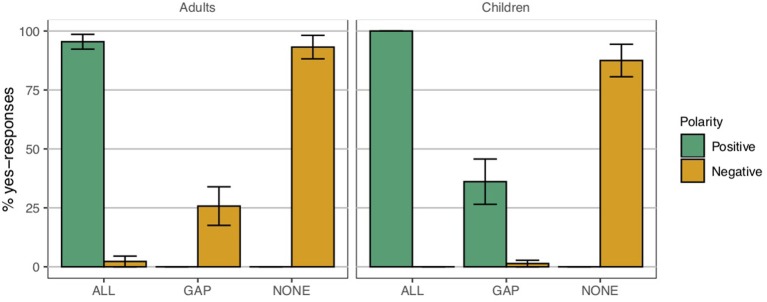
Percentage of *yes*-responses to the plural definite description targets and controls. Error bars correspond to the standard error of the within-participant means. Homogeneity targets corresponded to plural definite descriptions of gap contexts. Clearly true controls corresponded to positive plural definite descriptions of all contexts and negative plural definite descriptions of none contexts. Clearly false controls corresponded to positive plural definite descriptions of none contexts and negative plural definite descriptions of all contexts.

Figure 6 displays the percentage of *yes*-responses for the homogeneity targets, in between the true and false definite description controls. While children were adult-like with respect to the definite description controls, the two groups differed in their treatment of the definite plural in gap contexts. Unlike the adults, the children showed some acceptance of the positive definite descriptions in gap contexts; a mixed effects logistic regression model of responses as predicted by polarity revealed that they accepted the positive targets significantly more than they did the negative targets (*p* < 0.001) (lme4 package for R, R Core Team, 2016, Bates et al., 2015).

That children behaved differently from adults indicates that the child participants as a group were non-adult-like in their interpretation of the definite plural; but we wished to explore further *how* they might be interpreting the plural definite descriptions. Previous studies have hinted at existential, universal, and homogeneous possibilities, but these studies were inconclusive in this respect due to the absence of negative definite description targets. To further explore the possible interpretive preferences, we categorized participants according to their responses to both the positive and negative homogeneity targets. A participant was categorized as exhibiting the homogeneous response pattern if they rejected at least two of three positive homogeneity targets and at least two of three negative homogeneity targets. A participant was categorized as exhibiting the existential response pattern if they accepted at least two of three positive homogeneity targets, and rejected at least two of three negative homogeneity targets. Finally, a participant was categorized as displaying the universal response pattern if they rejected at least two of three positive homogeneity targets, and accepted at least two of three negative homogeneity targets^13^.

Table 1 displays the distribution of participants in the different response categories, based on their performance on the homogeneity and scalar implicature targets. Some readers would prefer an alternative analysis that does not bin participants into categories; we include this discussion here as an exploration of the possible interpretive profiles. As it turns out, our participants aligned rather strikingly into a subset of the possible categories. Let us first focus on the homogeneity targets. Sixteen of the 22 adult participants displayed the homogeneous pattern of responses, rejecting both positive and negative definite descriptions in gap contexts, while six adult participants displayed the universal response pattern, accepting the negative targets but rejecting the positive targets. Children treated the homogeneity targets differently from the adult group: sixteen of the 24 children displayed the homogeneous pattern of responses, while the remaining eight children displayed the existential response pattern (χ^2^(2, *N* = 46) = 13.94, *p* < 0.001). No adult displayed the existential response pattern and no child displayed the universal response pattern.

**Table 1:** Distribution of participants according to their performance on homogeneity and scalar implicature targets.

	**Adults**		**Children**	
	**− Implicature**	**+ Implicature**	**− Implicature**	**+ Implicature**
**Homogeneous**	5	10	6	10
**Existential**	0	0	7	1
**Universal**	5	1	0	0

We also elicited follow-up justifications following children's responses. The explanations that children provided indicate that they were generally consistent in their responses to the target conditions. Children consistently rejected the negative plural definite descriptions in gap contexts, justifying their answers by pointing out the objects that had the color mentioned by the puppet, as in (15).

(15) Justifications for rejecting negative homogeneity targets
  a. *Les camions ne sont pas bleus*. “The trucks are not blue
    ” CHI: (Non) parce qu'il y en a des bleus
    “(No) because there are blue ones” (C03-A, age 4;09,20)
  b. *Les camions ne sont pas bleus*. “The trucks are not blue
    ” CHI: (Non) parce que les camions ils sont bleus et jaunes
    “(No) because the trucks are blue and yellow” (C05-A, age 4;09,21)
  c. *Les balles ne sont pas rouges*. “The balls are not red” CHI: Pas vrai. Il y en a qui sont rouges
    “Not true. There are some that are red” (C07-B, age 4;11,19)

The *yes*-responses observed in the positive definite gap condition were primarily elicited from eight children who consistently accepted in this condition. These children justified their *yes*-responses by pointing out the objects that were of the color mentioned by the puppet, as in (16).

(16) Justifications for accepting positive homogeneity targets
  a. *Les ballons sont rouges*. “The balloons are red”CHI: (Oui) elle a dit qu'ils sont rouges
    “(Yes) she said they're red” (C02-B, age 4;04,15)
  b. *Les ballons sont rouges*. “The balloons are red”
    CHI: (Oui) parce qu'il y en a deux rouges
    “(Yes) because there are two red ones” (C03-A, age 4;09,20)
  c. *Les voitures sont bleues*. “The cars are blue”
    CHI: (Oui) elle a raison, elle a dit les voitures elles sont bleues
    “(Yes) she's right, she said the cars are blue” (C09-A, age 4;05,09)

The homogeneous children who rejected the positive homogeneity targets justified their responses by drawing attention to the objects that were of the color not mentioned by the puppet, as in (17).

(17) Justifications for rejecting positive homogeneity targets^14^
  a. *Les ballons sont rouges*. “The balloons are red”
    CHI: (Non) parce qu'ils sont rouges et bleus
    “(No) because they are red and blue” (C05-A, age 4;09,21)
  b. *Les étoiles sont jaunes*. “The stars are yellow”
    CHI: (Non) parce qu'elle a oublié les rouges !
    “(No) because she forgot the red ones” (C07-B, age 4;11,19)
  c. *Les voitures sont bleues*. “The cars are blue”
    CHI: (Non) c'est pas tout bleu
    “(No) it's not all blue”    (C12-B, age 4;07,14)

#### 4.2.2. Scalar Implicatures

Children's performance in the scalar implicature condition was comparable with that of the adult participants: children rejected the existentially quantified descriptions of all contexts 46% of the time, while adults did so 50% of the time. The distribution of adult and child participants according to their performance on homogeneity and scalar implicature targets is summarized in Table 1. An examination of individual children's responses in this condition revealed two groups of children: those who consistently failed to compute the implicature, accepting on at least three of four implicature trials, and those who consistently computed the implicature, rejecting on at least three of four implicature trials. Eleven of the 24 children consistently computed implicatures, providing justifications consistent with the strengthened meaning of the sentences:

(18) Justifications consistent with calculation of scalar implicature
  a. *Certains chapeaux sont roses*. “Some hats are pink”
    CHI: (Non) tous les chapeaux sont roses
    “(No) all of the hats are pink” (C10-B, age 4;10,12)
  b. *Certains chapeaux sont roses*. “Some hats are pink”
    CHI: (Non) parce qu'elle a dit certains […] j'aurais dit qu'ils sont tous roses
    “(No) because she said some […] I would have said they're all pink” (C11-A, age 5;00,05)
  c. *Certaines tentes sont oranges*. “Some tents are orange”
    CHI: Oh non, parce qu'elles sont toutes oranges
    “Oh no, because they're all orange” (C13-B, age 4;09,16)

In all, 13 of the 24 children failed to compute scalar implicatures, accepting on at least three of the four scalar implicature trials. Seven of these children were among the eight children who displayed the existential response pattern to the homogeneity targets, accepting the positive homogeneity targets and rejecting the negative ones^15^. The other six children who failed to compute implicatures were a subset of the 16 children who displayed the homogeneous response pattern.

#### 4.2.3. Non-randomness of Groupings

One potential concern about the groupings reported above is that some children, having not yet acquired the relevant construction, simply answered randomly (that is, at chance) on the homogeneity targets or the implicatures targets, or both, and therefore our diagnosis of a group of children with homogeneity but no implicatures may be spurious.

Recall that there were three items per condition and participants were categorized by their majority response. Based on the two homogeneity target conditions (the-some-pos and the-some-neg), every participant is thus guaranteed to fall into one of four possible groups. The fourth group was not mentioned in the preceding discussion because it turns out to be empty and is the least plausible from a theoretical point of view: it would correspond to interpreting the definite description as an existential that takes scope above negation. Now, given that two of the six possible groups are empty, it would be rather surprising if all six of the homogeneous/−implicature children ended up in this group by giving random responses, without any child ending up in one of the two empty groups (where they could have landed just as well by answering randomly).

To put a number on it, assume the following. Take children's answers on implicature targets to be non-random. This means we can exclude the +implicature children from consideration, since they cannot, in virtue of randomness of their responses to homogeneity targets, end up in the homogeneous/−implicature group. Now assume that of the 13 −implicature children, a certain number *n* answered randomly on homogeneity targets. Since the hypothesis is that the whole homogeneous/−implicature group is spurious, the value of *n* has to be at least 6. Now consider the probability, as a function of *n*, that the results would be at least as extreme as they actually are, in the following sense: at least six children are categorized as homogeneous/−implicature, and the other children are categorized as existential/−implicature, while the other two possible groups are empty. (The remaining 13 − *n* non-random responders fall in the existential/−implicature group in any case.) We find that for all values of *n* in [6, 13], with the exception of *n* = 8, *p* < 0.0005 (for *n* = 8, *p* < 0.001). Alternatively, assume that the random responders answered randomly on both homogeneity and implicature targets. We consider a result to be at least as extreme as ours if the following is the case: at least six children are in the homogeneous/−implicature group, no children are in the universal or the wide-scope existential group, and at most one child is in the existential/+implicature group. Then for any value of *n* in [6, 24], *p* < 0.0001.

**Figure 7:**
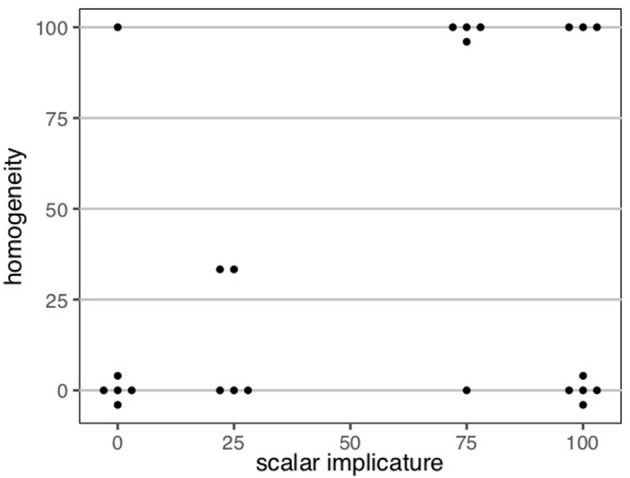
Within-subject mean responses on affirmative homogeneity and implicature items for children in Experiment 1 (recall that rejection (0) on the task corresponded to +homogeneous and +implicature responses).

To see the point in a more visual form, consider Figure 7, which shows individual children's mean responses to the positive homogeneity and implicature targets, with each data point corresponding to an individual child. The four corners correspond to groups: existential/+implicature in the upper-left, existential/−implicature in the upper-right, homogeneous/+implicature in the lower-left, and homogeneous/−implicature in the lower-right corner. Observe that children do, indeed, cluster into the corners nicely and the center of the plane is empty, indicating that children's responses are systematic and not random, legitimizing the binning into groups.

We may thus safely conclude that our finding is not an artifact created by children simply giving random responses to homogeneity targets coupled with a categorization rule that is based on the majority response in an odd number of trials.

#### 4.2.4. Improved Group Assignment

The purpose of this section is to provide a more solid underpinning for the descriptive categorization of participants we gave above. While in the previous section, we established that the observed group assignment is highly unlikely to be the result of purely random responses, our child data are clearly quite noisy, which a simple categorization based on majority response does not take into account. This issue will become especially pressing in Experiment 2, where the number of possible groups is much larger. We thus performed a categorization of participants on the basis of a statistical model of the responses.

The task is to assign a *group* to every participant, where there are 6 possible groups determining (i) whether or not the participant derives implicatures and (ii) what reading this participant assigns to definite descriptions (homogeneous, existential, universal). A group thus determines a theoretical response to each condition, as described in Table 2. We fitted logit models of the data (including both target and control conditions), with fixed intercept and slope and a subject-dependent group parameter as a predictor variable, varying by subject ^16^. The probability that a participant belongs to a given group is then given by the posterior probability of that value of the group parameter for that participant ^17^.

**Table 2:** Predicted responses to each condition ([determiner]-[context]-[polarity]) for each of the six possible groups, defined by the reading for the definite (existential, homogeneous, universal) and the presence or absence of implicatures.

	**exi**		**hom**		**uni**	
	**+si**	**−si**	**+si**	**−si**	**+si**	**−si**
the-all-neg	0	0	0	0	0	0
all-gap-pos	0	0	0	0	0	0
all-gap-neg	1	1	1	1	1	1
the-none-pos	0	0	0	0	0	0
some-all-pos	0	1	0	1	0	1
the-none-neg	1	1	1	1	1	1
the-gap-pos	1	1	0	0	1	1
the-gap-neg	0	0	0	0	1	1
the-all-pos	1	1	1	1	1	1

For children, the model fitted with all six possible levels for the group predictor indicated no mentionable posterior probability of a universal reading of the definite plural for any child. The two corresponding groups were thus subsequently dropped and the analysis was re-run with only four possible values for the group parameter. Children were assigned to groups quite unambiguously: the posterior probability of the group with the highest posterior probability ($\underset{g}{\max}p\left(\gamma \left(s\right)=g|Y\right)$) was >0.92 for all children and >0.99 for all except two. The result, shown in Table 3, replicates exactly our descriptive categorization^18^.

**Table 3:** Count of group assignments ($\arg\underset{g}{\max}p\left(\gamma \left(s\right)=g|Y\right)$) for children in Experiment 1.

	**−SI**	**+SI**
hom	6	10
exi	7	1

One might want to evaluate more directly whether the hom/−si and the exi/+si groups can be assumed to be populated. To do so, we compared models which made these groups a possibility with models which did not, using a leave-one-out cross-validation as recommended by Vehtari et al. (2017) ^19^. Table 4 summarizes the obtained estimated log pointwise predictive likelihoods. We see that models with the hom/−si group perform much better than those that do not include it (e.g., with all other groups included, Δ_elpd_ = 50.79 with *se* = 9.87), showing that this group is indeed populated. In comparison, models including the exi/+si group outperform their counterparts without it by only a small margin (e.g., Δ_elpd_ = 7.67 with *se* = 5.08).

**Table 4:** Estimated log pointwise predictive likelihood (elpd) and its standard error with different available group assignments for children.

**Possible groups**	**elpd**	**(se)**
All	−130.30	(15.86)
All but exi/+si	−137.97	(15.98)
All but hom/−si	−181.09	(15.90)
All but exi/+si and hom/−si	−189.79	(15.90)

“*All” groups were exi/±si and hom/±si*.

The model for adults was also first fitted with all six groups, followed by dropping the possibility of an existential reading since the model was found not to make use of it. Group assignment was again quite unambiguous^20^. The results are shown in Table 5; they are qualitatively comparable to our descriptive categorization from the previous section.

**Table 5:** Count of group assignments ($\arg\underset{g}{\max}p\left(\gamma \left(s\right)=g|Y\right)$) for adults in Experiment 1.

	**−SI**	**+SI**
hom	8	11
uni	3	0

### 4.3. Discussion

Let us first consider the results from the adult participants. Adult subjects were about equally split between those who did and those who did not derive scalar implicatures. This is not surprising given that implicatures are often said not to be obligatory^21^ and participants have previously been found to vary in the rate of implicature-based responses in such tasks (see e.g., Noveck and Posada, 2003).

As for the definite descriptions, the overwhelming majority of adults interpreted them homogeneously and none treated them as existential, as we would expect. In addition, a small number of participants treated the plural definite description like a (low-scope) universal; that is to say, in gap situations they judged affirmative the-sentences false, but negated ones true.

One can think of various possible explanations for this. One is that the definite description is really a universal for all speakers, but some chose the wide-scope and some chose the low-scope reading in a scopally ambiguous case, such as that of sentential negation. Since, however, there are independent arguments for why homogeneity is not simply universally interpreted definite plurals taking wide scope^22^, this has little plausibility.

Alternatively, these particular participants might just have a different understanding of the definite from the majority, namely a universal as opposed to a homogeneous one. This hypothesis would be quite testable precisely on the basis of the arguments for a distinction between homogeneity and wide-scope universals, since these participants would be predicted to behave distinctly on such cases. However, we do not pursue this question further here.

Finally, these participants might be employing a different response strategy: instead of first computing the truth value of the sentence in a trivalent setting and then mapping these three truth values to two truth values to generate their response, they might, following the intuition that negation should invert the truth value, first compute their response for the positive sentence and then simply reverse it to obtain the response for the negated sentence^23^.

Turning to the children's responses, recall that the first goal of the experiment was to resolve the uncertainty surrounding the interpretations that young children assign to plural definite descriptions; previous studies had hinted at existential, universal, and homogeneous possibilities, but these hints were inconclusive due to the absence of the negative counterparts. The results of Experiment 1 revealed two groups of children, based on responses to both positive and negative definite descriptions: one group interpreted the definite descriptions existentially (scoping under negation), while the other interpreted them homogeneously. We had initially reasoned that a universal interpretation would be plausible on the basis of considerations of the input. If a child were to hear positive plural definite descriptions exclusively in scenarios that satisfied homogeneity, for example, that could be a strong reason to posit a universal meaning for the definite plural. The fact that no child displayed the universal pattern of response, however, suggests this is not the case. Instead, children might be led to posit an existential meaning for the definite plural, on the basis of its behavior under negation, and the occasional non-maximal reading of the definite plural (for discussion of non-maximal readings, see Brisson, 1998; Lasersohn, 1999; Malamud, 2012; Schwarz, 2013; Križ, 2015a).

Note another important finding of Experiment 1. While non-maximal responses from children could be argued to arise from non-adult-like domain restriction, the inclusion of negative targets in our experiment allows us to rule out such an explanation for their seemingly existential readings of plural definite descriptions. If children (in our experiment as well as in the previous experiments we've discussed) were to accept the positive plural definite descriptions in a gap scenario because they restricted the domain to the individuals that did indeed satisfy the predicate, one would expect them to be able to accept the negative homogeneity targets using an analogous strategy of restricting the reference to those individuals who do not satisfy the predicate. In essence, such ‘wildly domain-restricting' children would interpret the positive and negative homogeneity targets as in (19) and (20), respectively.

(19) The hearts are red.
  ⇝ The hearts that are red are red.
(20) The hearts are not red.
  ⇝ The hearts that are not red are not red.

The fact that the children we tested, in particular those who accepted the positive homogeneity targets, never accepted the negative targets, suggests that acceptance of homogeneity violations cannot be due to non-adult-like domain restriction.

The second goal of the experiment was to investigate the predictions of the scalar implicature account of homogeneity. On this account, the definite plural has a literal existential meaning, which is then strengthened to a universal meaning through an implicature. The finding of an existential subgroup of children, who moreover lacked scalar implicatures, is consistent with and expected on the implicature account of homogeneity. Unable to derive the homogeneous meaning through implicature, these children start out with judgments based on the literal, existential meaning of the definite plural.

The implicature account also makes the further prediction, however, that homogeneity should not be observed in the absence of scalar implicatures. This prediction comes in two parts. First, children who have not yet acquired scalar implicatures should be unable to obtain homogeneous readings for plural definite descriptions. Second, the scalar implicature from *some* to *not all* should not occur at a lower rate than homogeneous readings because this implicature is actually a subcomputation of the homogeneity implicature in Magri's theory. If anything, homogeneity should occur at a lower rate than the regular scalar implicature.

Even among our adult participants, roughly half were categorized as not computing implicatures. This means that we cannot conclude that the children who are categorized as not deriving implicatures have indeed not yet acquired them, since it is also possible that they simply refrain from computing implicatures for the same reason that some of the adults do. Consequently, our data do not speak to the first prediction of the implicature theory. The second prediction, however, is clearly falsified for both children and adults: in both groups, the failure to derive scalar implicatures is more prevalent than non-homogeneous interpretations of plural definite descriptions^24^. Most strikingly, there was no group of participants who systematically derived scalar implicatures and at the same time failed to access homogeneous readings of definite plurals. This suggests that there is, in fact, an alternative way of obtaining homogeneous readings that does not rely on scalar implicatures, and that this alternative way of generating homogeneity is already acquired by the time children are robustly computing scalar implicatures.

A remaining worry is that our diagnosis of universal and homogeneous readings might be confounded by the scope of negation. The present analysis is predicated on the assumption that the definite plural, whatever its meaning, takes low scope under sentential negation. However, in order to keep the sentences and visual display simple, we had the definite plural in the subject position of intransitive sentences, which means that its surface scope was actually above sentential negation. If children interpreted the definite plural as a universal in surface scope position, i.e., with wide scope over negation, then that would give rise to the same responses as a homogeneous meaning in our binary judgment paradigm: both affirmative and negative sentences with definite plurals would be judged false (i.e., non-true) in gap situations. As there is not much of a difference in either the mean or youngest age of participants in the existential vs. the homogeneous group (mean 4.72 years and minimum 4.37 years vs. mean 4.73 years and minimum 4.42 years), it is possible that some children start out with a (low-scope) existential reading and others start out with a (wide-scope) universal reading for the definite plural. Experiment 2 is an attempt to control for this possibility.

Note that a wide-scope universal is not under discussion as a possible reading of the definite plural for adults. This reduces the plausibility of the above worry for children, and makes it entirely inapplicable to our argument against the implicature theory on the basis of the adult data.

## 5. Experiment 2

The goal of Experiment 2 was to obtain a more fine-grained picture in which truly homogeneous readings would be distinguished from wide-scope universals. In order to do this, what we require is a way to distinguish merely non-true sentences from those that are *bona fide* false, to which end a ternary response paradigm has been employed for adults by Križ and Chemla (2015). A ternary response paradigm has also been used with children in an investigation of scalar implicatures. Katsos and Bishop (2011) report that when given the choice between a minimal, an intermediate, and a maximal reward option, 5-year-old children are adult-like in consistently choosing to give the puppet the intermediate reward for a literally true utterance with a false implicature. We were thus hopeful that a similar implementation of the ternary response paradigm would allow us to shed further light on the interpretations that children assign to plural definite descriptions.

### 5.1. Methods

Ethical approval for this study was obtained from the CERES (“Comité d'évaluation éthique des projets de recherche en santé non soumis à CPP”) under approval number 2013/46. Written informed consent was obtained from the parents or guardians of all child participants; adult participants were tested through an anonymous web-based survey, and had to click a button to provide informed consent before starting the experiment.

#### 5.1.1. Participants

We tested 24 French-speaking children (10 female) (4;07, 04 − 6;04, 13, *M* = 5;03) at a preschool in Paris. Three additional children did not finish the task, and another two were excluded from analysis because they answered fewer than six of eight control trials correctly (using the same control trials as in Experiment 1, in which a sentence with a definite description was made uncontroversially true or false). We also tested 25 adult native speakers of French, recruited through the online platform FouleFactory, at a total cost of € 38.30. All adult participants passed the controls and were included in the analysis.

#### 5.1.2. Procedure

**Figure 8:**
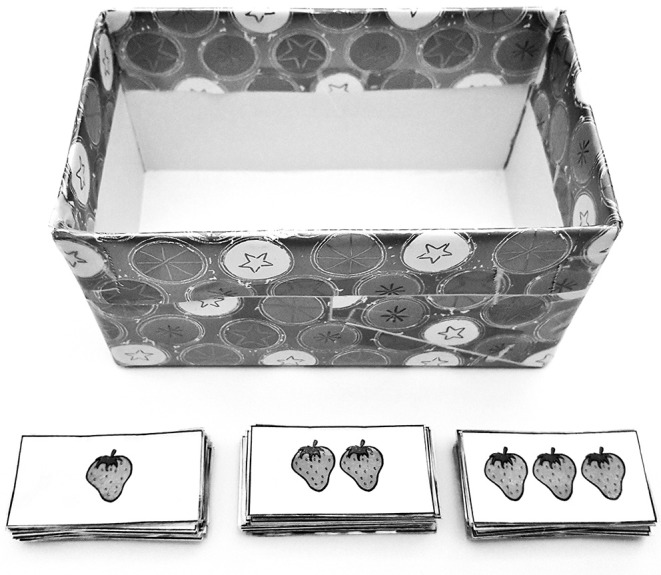
Materials used in the ternary judgment task. Clearly false targets were meant to elicit the minimal reward of one strawberry. Clearly true targets were meant to elicit the maximal reward of three strawberries. Based on the results reported in Katsos and Bishop (2011), the intermediate reward of two strawberries was meant to correspond to underinformative targets.

Children were introduced to Boba the puppet, who interacted via webcam. Children were told that Boba was still very little, and not very good at paying attention. Children were then presented with a series of pictures on a laptop computer, each containing four objects, just as in Experiment 1. They were asked to identify the colors of each of the four objects. The puppet was then asked to say something about the objects, and would utter a test sentence containing a plural definite description (e.g., *les ballons* “the balloons”), an existentially quantified noun phrase (e.g., *certains ballons* “some balloons”), or a universally quantified noun phrase (e.g., *tous les ballons* “all the balloons”). Children had to decide whether the puppet's description was worth a reward of one, two, or three strawberries. Children indicated their choices by choosing cards with the appropriate number of strawberries on them and placing them in a box in front of the laptop (Figure 8).

Although children in this age range have been reported to engage quite naturally with these kinds of graded reward scales (Katsos and Bishop, 2011), some time at the beginning of the experiment was devoted to making sure each child understood how to use the scale. The instructions for each child included an explanation of how to use the graded reward scale, and the child was encouraged to explain back to the experimenter what each reward meant, to make sure they had understood. Only once the child showed a solid understanding of the three possible rewards did the task begin. The instructions are provided in the Appendix, in both French and English.

Children were tested individually away from their classrooms. Responses were videorecorded for subsequent analysis. Children saw two training items containing single objects (e.g., a pink chair), followed by 26 test trials presented in one of two pseudorandomized orders (the reverse of each other). The total task took roughly 10–15 min for each child to complete.

Adults were tested on a web-based version of the task; sentences were presented visually in the form of speech bubbles, and adults indicated their responses by clicking on appropriate buttons depicting the three reward options.

#### 5.1.3. Materials

The materials used in Experiment 2 took essentially the same form as those in Experiment 1, but some additional control conditions were required because of the nature of the judgment task. Recall that the primary goal of this experiment was to tease apart homogeneous readings of definite descriptions from wide-scope universals by giving participants an intermediate response option that could be used to indicate a homogeneity violation. This is complicated by the fact that an intermediate response to a homogeneity target may conceivably arise for any of the following reasons:

(21) Possible sources of an intermediate reward for a positive homogeneity target, e.g., *The hearts are red* in a gap context
  a. The child interpreted the definite description homogeneously.
  b. The child interpreted the definite description existentially (*Some of the hearts are red*), but didn't want to give the maximal reward because the sentence was an incomplete description of the image.
  c. The child interpreted the definite description universally (*Every heart is red*), but didn't want to give the minimal reward because the sentence was a true description of at least part of the image.

Likewise, an intermediate reward for a negative homogeneity target would ideally reflect a child's sensitivity to the violation of homogeneity. But it could arise for any of the reasons in (22).

(22) Possible sources of an intermediate reward for a negative homogeneity target, e.g., *The hearts are not red* in a gap context
  a. The child interpreted the definite description homogeneously.
  b. The child interpreted the definite description existentially (*Some of the hearts are not red*), but didn't want to give the maximal reward because the sentence was an incomplete description of the image.
  c. The child interpreted the definite description universally (*Every heart is not red*), but didn't want to give the maximal reward because the sentence was true on only one of the two possible scopal construals.

To address these potential confounds, we included three kinds of controls in this experiment: *incomplete description* existential controls, *partial truth* universal controls, and *scope ambiguity* universal controls. If a child did not give intermediate responses in these conditions, then we could exclude these three confounds as potential explanations for intermediate responses to the homogeneity targets. The specific sentence types used to control for these three confounds will be described in the appropriate sections below, alongside the corresponding target sentence types.

***Plural definite descriptions***. Experiment 2 included positive and negative sentences containing plural definite descriptions, as in (23). They were combined with different types of situations (pictures) to form homogeneity targets, as well as clearly true and clearly false controls.

(23) a. Les coeurs sont rouges.
  “The hearts are red.”
b. Les coeurs ne sont pas rouges.
  “The hearts are not red.”

**Figure 9:**
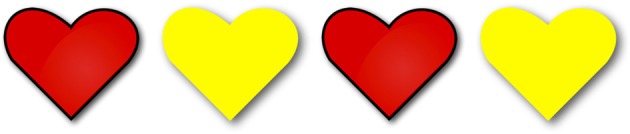
Image corresponding to a gap context. The first and third hearts are red, while the second and fourth hearts are yellow. If used on a homogeneity target trial, this image would accompany either the positive *Les coeurs sont rouges* “The hearts are red” or the negative *Les coeurs ne sont pas rouges* “The hearts are not red.” If used on an incomplete description control trial, this image would accompany the sentence *Certains coeurs sont rouges* “Some hearts are red.” If associated with a partial truth control, this image would accompany the positive *Tous les coeurs sont rouges* “All the hearts are red.” Finally, if associated with a scope ambiguity control, this image would accompany the negative *Tous les coeurs ne sont pas rouges* “Not all the hearts are red.”

Participants received three positive and three negative homogeneity target trials. On these target trials, they had to judge positive and negative *les* “the”-NP sentences such as (23), presented in gap contexts in which only two of the four objects in the image were of the color indicated in the test sentence (Figure 9).

**Figure 10:**
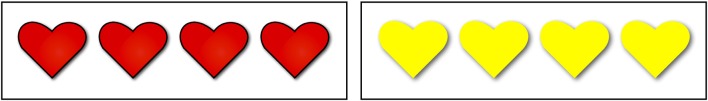
Images corresponding to the clearly true and clearly false definite plural controls. When accompanied by the all context image on the left, in which all four hearts are red, the positive and negative descriptions in (23) would be associated with a maximal reward target and a minimal reward target, respectively. When accompanied by the none context image on the right, in which all four hearts are yellow, the positive and negative sentences in (23) would be associated with a minimal reward target and a maximal reward target, respectively.

Participants also received four clearly true or clearly false positive definite description controls, and four clearly true or clearly false negative definite description controls. On these control trials, participants heard sentences containing plural definite descriptions just like (23), but presented in contexts that satisfied homogeneity, i.e., where all four objects displayed were of the same color (Figure 10). In all contexts, where all of the objects shared the color indicated in the test sentence, the positive control (23a) was associated with a maximal reward target, and the negative control (23b) with a minimal reward target. In none contexts where none of the objects had the color indicated in the test sentence, the positive definite description (23a) was associated with a minimal reward target, and the negative (23b) with a maximal reward target.

Whether a definite plural control sentence was accompanied by an all or a none picture was determined dynamically, on the basis of children's responses to the target trials^25^. This allowed us to avoid eliciting overly long sequences of the same response (for example, a string of successive intermediate rewards), which otherwise could have encouraged a biased response strategy. These controls also allowed us to ascertain that children understood definite descriptions, and in particular could provide minimal and maximal reward judgments appropriately when there were no issues of non-homogeneity. Any participant who failed to correctly answer at least six of the eight definite plural controls was excluded from analysis.

***Existential quantification conditions***. Experiment 2 also contained positive existentially quantified sentences such as (24). They were combined with two types of situations (pictures) to form scalar implicature targets and incomplete description controls.

(24) Certains coeurs sont rouges.
  “Some hearts are red.”

On scalar implicature trials, participants heard such sentences in contexts where all four objects displayed were of the mentioned color. Each participant received three such trials. As with the homogeneity targets, we expected that if participants computed the scalar implicatures, they would opt to give either minimal or intermediate rewards, but not maximal rewards. This is because although the sentences are true on their literal meaning, the context falsifies the associated scalar implicatures. Previous work by Katsos and Bishop (2011) suggests that children are likely to give intermediate rewards for such cases of underinformative descriptions.

On the *incomplete description* controls, participants heard existentially quantified sentences as descriptions of gap contexts. For example, they would hear a sentence like (24), accompanying the image in Figure 9^26^. These sentences are uncontroversially true in such contexts, but they do not offer a complete description of the situation and here quite visibly so: a color present in the picture is not at all mentioned in the sentence. So if a participant gives an intermediate reward on these control trials, we may suspect that other intermediate rewards they might give for homogeneity targets could also be due to incomplete description effects. Each participant received three repetitions of this control.

***Universal quantification conditions***. Finally, Experiment 2 also contained positive and negative universally quantified sentences, as in (25). These were combined with gap contexts to form partial truth controls and scope ambiguity controls.

(25) a. Tous les coeurs sont rouges.
  “All the hearts are red.”
b. Tous les coeurs ne sont pas rouges.
  “All the hearts are not red.”
  Intended interpretation: “Not all the hearts are red.”^27^

On partial truth controls, positive universally quantified sentences such as (25a) were presented in gap contexts like Figure 9, in which only two of the four objects were of the color indicated in the test sentences. Each participant received three such trials. These sentences were uncontroversially false in gap contexts, so if a participant gave an intermediate reward rather than a minimal reward, we could reasonably infer that they had a bias for rewarding the puppet for having given a truthful description of at least part of the picture. This would then give us reason to suspect that any intermediate responses the participant may have given on the homogeneity targets could also have arisen from these partial truth effects.

On scope ambiguity controls, a negative universally quantified sentence such as (25b) was presented in a gap context like Figure 9. Each participant received three such trials. On the intended interpretation, the negative sentences were true in gap contexts. On the other construal, on which the universal scopes above negation, the sentences were false. If a child gave an intermediate reward rather than a maximal reward, this could reflect a dispreference against sentences that had at least one false reading. In other words, the puppet would receive a reward for saying something that had a true reading, but would not receive the maximal reward because the utterance was not *unambiguously* true. This would then give us reason to suspect that any intermediate responses the participant may have given on the negative homogeneity targets could also have been given on the grounds of a scope ambiguity between a (universally or existentially interpreted) definite description and negation^28^.

***Summary of the materials***. In all, participants received two training items, six homogeneity targets, eight uncontroversially true/false plural definite description controls, three scalar implicature targets, three incomplete description controls, three partial truth controls, and three scope ambiguity controls. The full set of test sentences is provided in the Appendix.

### 5.2. Results

#### 5.2.1. Existential Quantification Conditions

**Figure 11:**
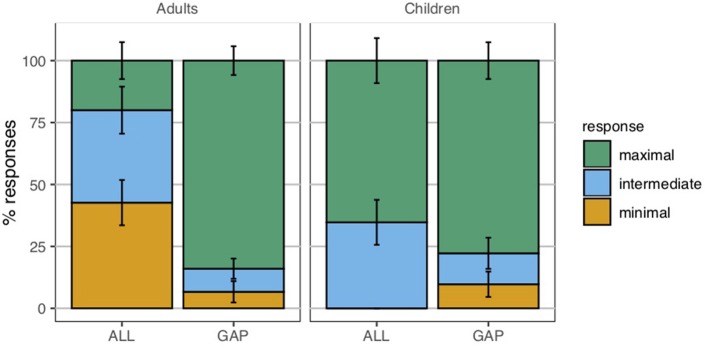
Percentages of the reward types given in the existential *certains* “some” conditions. The all context corresponded to the scalar implicature targets, and the gap context corresponded to the *incomplete description controls*. Minimal or intermediate rewards for existentially quantified sentences in all contexts were indicative of scalar implicatures. A less-than-maximal reward for existential descriptions of gap contexts was indicative of incomplete description effects.

Figure 11 displays the percentages of the reward types given in the existential quantification conditions. In response to the *scalar implicature targets*, i.e., existentially quantified sentences in all contexts, children gave more maximal rewards than adults, suggesting they computed fewer scalar implicatures than adults did. They also never gave *minimal* rewards on the basis of a false implicature and were thus, in a sense, more forgiving than adults. In response to the *incomplete description controls*, i.e., existentially quantified sentences in gap contexts, children performed on a par with adults, generally maximally rewarding the puppet. This suggests that incomplete description effects do not play much of a role: children did not appear to be less inclined to give a high reward simply because the puppet had not described all of the objects in the picture.

#### 5.2.2. Universal Quantification Conditions

**Figure 12:**
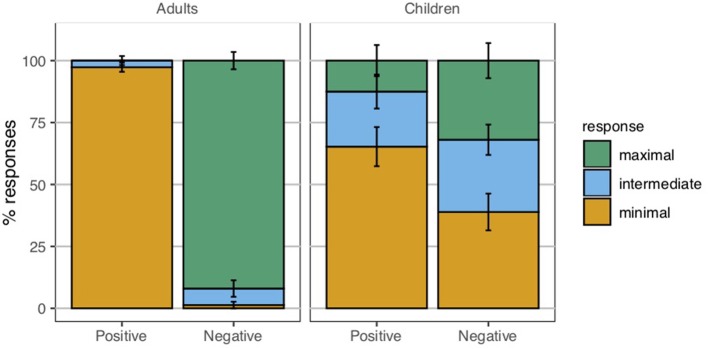
Percentages of the reward types given in the universal *tous* “all” conditions. Positive universal descriptions of gap contexts corresponded to *partial truth* controls; negative universal descriptions of gap contexts corresponded to *scope ambiguity* controls. A greater-than-minimal reward for positive universal descriptions of gap contexts was indicative of partial truth effects. A less-than-maximal reward for negative universal descriptions of gap contexts was indicative of scope ambiguity effects.

Figure 12 displays the percentages of the reward types given in the universal quantification conditions. In response to the *partial truth controls*, i.e., positive universally quantified descriptions of gap contexts, both adults and children gave less-than-intermediate rewards. The fact that children gave fewer minimal rewards than adults in this condition could be suggestive of a tendency to reward for partial truth.

In response to the *scope ambiguity controls*, i.e., negative universally quantified descriptions of gap contexts, adults predominantly gave maximal rewards, which means that they interpreted the universal as scoping under negation. Children, on the other hand, were quite varied in their responses. While the maximal and minimal responses correspond to one of the readings of the sentence, intermediate responses may have two explanations. First, the intermediate rewards could reflect recognition of a sentence that may be construed as true, but is not unambiguously so. Second, some children could have accessed the surface scope interpretation of the negative sentences (*All of the hearts are such that they are not red*) and rewarded the partial truth of this sentence (which is literally false) with an intermediate response. Given the magnitude of the proportion of intermediate responses in this condition compared to the *partial truth* controls, however, it seems implausible that the latter could be solely responsible. Thus, it is plausible that scope ambiguity in itself would sometimes give rise to intermediate responses.

#### 5.2.3. Plural Definite Description Conditions

**Figure 13:**
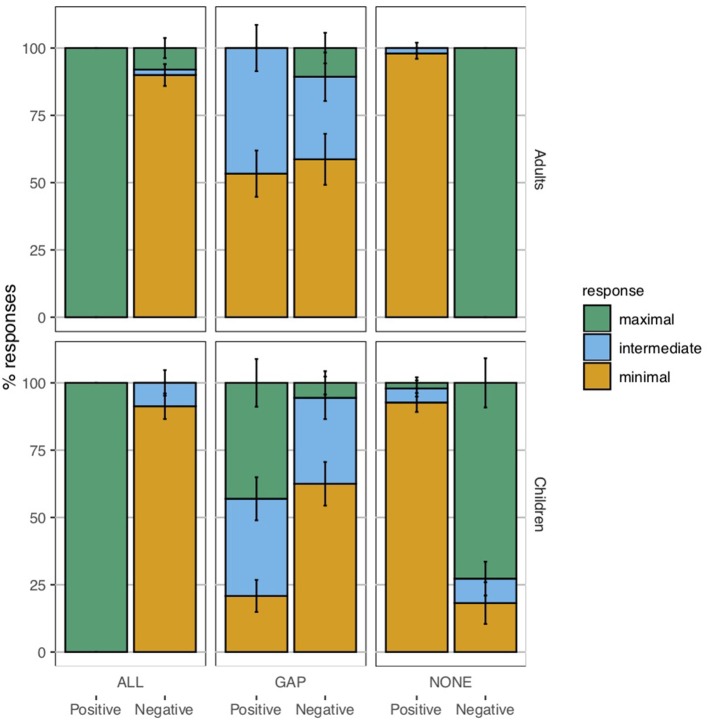
Percentages of the reward types given in the definite *les* “the” conditions. True controls corresponded to positive plural definite descriptions of all contexts and negative plural definite descriptions of none contexts. False controls corresponded to positive plural definite descriptions of none contexts and negative plural definite descriptions of all contexts. Homogeneity targets corresponded to plural definite descriptions of gap contexts.

Figure 13 displays the percentages of the reward types given in the plural definite description conditions. Adults and children generally performed as expected in three of the four unambiguous definite plural control conditions. In particular, they gave maximal rewards for the positive definite descriptions in all contexts (a *true* control) and minimal rewards for the positive definite descriptions in none contexts (a *false* control). They also gave minimal rewards for the negative definite descriptions in all contexts (a *false* control). In response to the negative definite descriptions in none contexts (a *true* control), however, children did not reward as maximally as adults did. A closer examination of children's responses and justifications suggests this was because children did not like the fact that the puppet's sentence mentioned a color that none of the objects shared. In other words, they may have seen some degree of infelicity associated with describing what color the objects were *not*, as opposed to what color they were^29^.

As for the homogeneity targets, children and adults again differed in their treatment of plural definite descriptions in gap contexts. First, as seen in Figure 13, adults generally gave the same responses to positive and negative homogeneity targets, while children tended to give greater rewards for positive homogeneity targets than for negative homogeneity targets. Second, the two groups differed in the distribution of individual participants across the different response categories. Participants were categorized as existential if they gave the maximal reward on at least two of three positive target trials, and if they gave the minimal reward on at least two of three negative target trials. Participants were characterized as homogeneous if they gave minimal or intermediate rewards on at least two of three positive and two of three negative target trials. Finally, participants were categorized as universal if they gave the minimal reward on at least two of three positive target trials, and if they gave the maximal reward on at least two of three negative target trials.

Table 6 represents the distribution of children and adults according to their performance on the homogeneity and scalar implicature targets^30^. Focusing first on the homogeneity targets, it is apparent that children and adults differed: while 23 of the 25 adults responded in a manner consistent with homogeneity, i.e., giving minimal or intermediate rewards to both positive and negative definite descriptions in gap contexts, 12 children (mean age 5;08) displayed this adult pattern and 10 children (mean age 5;00) displayed the existential response pattern, maximally rewarding the positive descriptions but minimally rewarding the negative descriptions (χ^2^(2, *N* = 47) = 15.33, *p* < 0.001). Two other children gave inconsistent responses.

**Table 6:** Distribution of participants across response types, according to performance on homogeneity and scalar implicature targets.

	**Adults**		**Children**	
	**− Implicature**	**+ Implicature**	**− Implicature**	**+ Implicature**
**Homogeneous**	2	21	5	7
**Existential**	0	0	10	0
**Universal**	2	0	0	0

*Two children gave inconsistent responses and are not included in the table*.

Returning to the full distinctions presented in Table 6, we can discuss the individual responses to both homogeneity and scalar implicature targets together. We observe the same two subgroups of children as in Experiment 1: a subgroup of existential children who failed to compute scalar implicatures, and a subgroup of homogeneous children, only some of whom computed implicatures. As in Experiment 1, no child displayed the universal response pattern.

Finally, we took into account the *incomplete description, partial truth*, and *scope ambiguity* controls, in order to completely factor out these potential biases as described above. Recall that each participant received three repetitions of each kind of control. A participant was considered to have a bias against incomplete descriptions if they gave the maximal reward on fewer than two of the three trials. A participant was considered to have a bias in favor of partial truth if they gave the minimal reward on fewer than two of the three trials. Finally, a participant was considered to display a scope ambiguity effect if they gave the intermediate response on more than one of the three trials.

In Table 7 we present the distribution of participants who passed this maximally conservative inclusion criterion. The remaining 21 adults and 9 children are those who we can be reasonably certain responded to the plural definite descriptions without any interfering or irrelevant biases. As was the case before the exclusions, we observe mostly homogeneous adults, and a homogeneous subgroup and an existential subgroup for children.

**Table 7:** Distribution of participants across response types, after applying a maximally stringent exclusion criterion that eliminated any participants who could potentially have had a bias for partial truth, against incomplete description, or against scope ambiguity.

	**Adults**		**Children**	
	**− Implicature**	**+ Implicature**	**− Implicature**	**+ Implicature**
**Homogeneous**	2	18	2	2
**Existential**	0	0	5	0
**Universal**	1	0	0	0

#### 5.2.4. Non-randomness of Groupings

Since the ternary judgment task involves three response options, the number of logically possible groups, defined by how often a participant chose which option on which of the three relevant conditions (the two homogeneity targets the-some-pos and the-some-neg, and the implicature target some-all-pos), is 27. Nevertheless, 22 of the 24 children fall into only three of these groups, and it is precisely the groups which, from a theoretical point of view, correspond to the three groups in which 23 of the 24 children were found in Experiment 1. It is thus highly unlikely that the five participants in the homogeneous/−implicature group are there simply by virtue of giving random responses. Since the relevant *p*-values are guaranteed to be much lower than even for Experiment 1 (section 4.2.3), we do not calculate them here.

#### 5.2.5. Improved Group Assignment

The purpose of this section is, again, to obtain a quantitative assessment of the preceding characterization of the data in terms of assigning children to groups. The question we are interested in is whether there is evidence for the existence of children with truly homogeneous interpretations for definite plurals but who do not compute implicatures. To this end, we will describe an analysis that allows us to decide for each participant whether they have implicatures and what reading they assign to the definite plural. The possible readings for the definite plurals that we consider are the following:

exi Low-scope existential interpretation for definite plurals (as before).
hom Truly homogeneous interpretation, which should lead to an intermediate response in both positive and negative the-gap conditions.
sa Universal interpretation with scope ambiguity effects, which should lead to a minimal response in the the-gap-pos condition since there is no possibility for scope ambiguity here, but to an intermediate response in the the-gap-neg condition because the sentence is either true or false depending on where the universal takes scope with respect to negation.
ws A strictly wide-scope universal interpretation, which should yield minimal responses in both positive and negative the-gap conditions.
pt Wide-scope universal interpretation with partial truth effects, which should yield intermediate responses in both the-gap conditions, like hom, but should additionally yield an intermediate response in the all-gap-pos condition (where hom would yield a minimal response).

We thus obtain in principle 5(exi, hom, sa, ws, pt) × 2(+si,−si) possible groups of participants. Each of these groups corresponds to a unique pattern of responses to the different conditions, as described in Table 8. The upcoming analyses fit ordinal regression models which assign each participant to a given group, given this participant's actual responses ^31^. The models may allow for different groups to be considered, and in order to decide whether it is meaningful to say that some participants belong to a particular group, we ask whether models that include that group are superior to models that do not include that group, all else being equal. One problem is that it is not necessarily possible to reliably estimate the relevant models with the whole dataset while considering all possible groups at once, so below we propose several analyses which are essentially similar but differ in what assumptions they rely on to simplify this computational limitation. In all these analyses, we rely on the results of Experiment 1, where only a single child was categorized as belonging to the group exi/+si, in not including that group in any of the models.

**Table 8:** Predicted responses to each condition ([determiner]-[context]-[polarity]) for each of the possible groups.

	**exi**		**hom**		**sa**		**ws**		**pt**	
	**+si**	**-si**	**+si**	**-si**	**+si**	**-si**	**+si**	**-si**	**+si**	**-si**
all-gap-neg	1	1	1	1	1	1	0	0	0	0
some-gap-pos	1	1	1	1	0	0	1	1	1	1
the-all-neg	−1	−1	−1	−1	−1	−1	−1	−1	−1	−1
the-gap-neg	−1	−1	0	0	−1	−1	0	0	0	0
the-none-pos	−1	−1	−1	−1	−1	−1	−1	−1	−1	−1
the-gap-pos	1	1	0	0	1	1	1	1	0	0
the-none-neg	1	1	1	1	1	1	1	1	1	1
the-all-pos	1	1	1	1	1	1	1	1	1	1
some-all-pos	0	1	0	1	0	1	0	1	0	1
all-gap-pos	−1	−1	−1	−1	−1	−1	−1	−1	0	0

***Analysis 1: no partial truth, implicatures imply homogeneity***

In this analysis, we restricted the dataset to the conditions with the plus the implicature-relevant condition some-all-pos. There is little reason to think that partial truth was playing any role and, accordingly, this analysis does not consider the possibility of a pt group (the role of pt groups is evaluated separately in Analysis 3). Furthermore, we assume here that every child who had acquired implicatures had also reached an adult-like stage for homogeneity. The only +si group allowed in these models was thus the hom/+si group. Apart from these restrictions, the models in this analysis explore all combinations of hom/−si, sa/−si, and ws/−si ^32^.

Table 9 shows the estimated log pointwise predictive likelihoods (elpd) and their standard errors for each of these models. Overall, models that included the hom/−si group were superior to those that did not, providing evidence in favor of the existence of a group of children with access to homogeneous readings but not to implicatures ^33^.

**Table 9:** Results of leave-one-out cross-validation for Experiment 2.

			**Analysis 1**		**Analysis 2**		**An. 1** ***vs*****. An. 2**		**Analysis 3**	
**hom/−si**	**ws/−si**	**sa/−si**	**elpd**	**(se)**	**elpd**	**(se)**	**Δ_elpd_**	**(se)**	**Δ_elpd_**	**(se)**
✓	✓		−238.9	(18.0)	−239.6	(18.2)	0.8	(3.6)	8.9	(4.3)
✓		✓	−240.6	(17.6)	−244.8	(17.8)	4.1	(3.8)	8.8	(4.5)
✓	✓	✓	−241.4	(17.9)	−241.6	(18.2)	0.3	(3.6)	8.3	(4.4)
✓			−241.5	(17.5)	−249.0	(17.7)	7.5	(4.6)	9.9	(4.7)
	✓		−247.7	(18.6)	−249.3	(18.7)	1.6	(3.8)	1.2	(6.9)
	✓	✓	−248.7	(18.5)	−249.8	(18.7)	1.1	(3.7)	1.6	(6.8)
		✓	−256.5	(18.2)	−253.0	(18.2)	−3.5	(5.2)	1.6	(6.9)

*All models included groups hom/+si and exi/-si on top of those marked in the table. Models in Analysis 2 additionally included sa/+si and ws/+si. Models in Analyses 1 and 2 were fitted on the conditions plus some-all-pos. Models in Analysis 3 were fitted on the same data plus all-gap-pos. The column for Analysis 3 shows the comparison of models that included the pt/−si with models that did not (positive numbers favor the latter)*.

***Analysis 2: no partial truth, no assumption that implicatures imply homogeneity***

This analysis differed from Analysis 1 in that it did not assume that implicatures imply homogeneous readings; that is, the groups sa/+si and ws/+si were systematically included in all models as possible groups a child could fall in. The overall picture remains largely the same, with elpds in the same range as in Analysis 1 (Table 9), and favoring models making use of the hom/−si group.

A comparison of the models from Analysis 2 to the corresponding models from Analysis 1 (also provided in Table 9) reveals that those from Analysis 1 actually perform better, suggesting that the assumption in Analysis 1 that homogeneity is systematically acquired earlier than implicatures is warranted^34^^,^^35^.

***Analysis 3: the role of partial truth***

For Analysis 3, we are interested in evaluating the role of the partial truth strategy. The target is thus the comparison of models with and without pt groups. Given the results of Analysis 2, we start over from Analysis 1, assuming that homogeneity precedes implicatures, i.e., dropping all +si group except hom/+si. In Analysis 3, the condition all-gap-pos was included alongside the conditions used in Analysis 1, because it is now necessary to differentiate the newly added pt/−si group from the hom/−si group. The last column of Table 9 presents a comparison of models with a partial truth pt/−si group with the corresponding models without such a group. The comparison uniformly comes out in favor of the models without pt/−si. Hence, this analysis provides no evidence for the existence of the pt/−si group or, to put it differently, in favor of the partial truth strategy.

### 5.3. Summary

The results of Experiment 2 replicate the essential findings of Experiment 1 insofar as, if one were to collapse intermediate and minimal rewards in the ternary paradigm, the resulting picture is very similar to what we saw in Experiment 1 on all the crucial points. Furthermore, we find that even if some of the children who do not compute implicatures may have a wide-scope universal reading for the definite plural (which Experiment 1 could not distinguish from a truly homogeneous one), there is evidence for a group with homogeneous readings and, nonetheless, no implicatures.

## 6. Discussion

The results of our experiments revealed, by and large, three groups of children. The first group of children did not compute implicatures and interpreted definite plurals as existentials (that scope under negation). A question that is raised by this state of affairs is the following. Children are evidently able to reach truth conditions equivalent to those of adults for negated sentences by recognizing that definite plurals, interpreted existentially, have to scope under negation. But why would they hypothesize an existential meaning in the first place when it results in truth conditions for affirmative sentences that are so different from those of adults? We can only offer some speculation as to how this asymmetry might come about. It is well-known that sentences with definite plurals are not infrequently used when there are some exceptions, even though under scrutiny we would not judge such sentences as strictly true. This phenomenon is known as *non-maximality* (Brisson, 1998; Lasersohn, 1999; Malamud, 2012; Križ, 2015b). An example from Lasersohn (1999) is (26), which can be felicitously used to describe a situation in which there are nevertheless a few insomniacs who are reading in bed and not actually asleep.

(26) The townspeople are asleep.

While the exceptions that can be ignored by way of non-maximality are typically few in number, in the right contexts, non-maximal readings can effectively turn existential, such as in this example from Malamud (2012):

(27) Context: *Mary has a large house with over a dozen windows in different rooms. She locks up and leaves to go on a road trip with her friend Max, forgetting to close just a few of the many windows in various rooms. A few minutes into the ride, Max says, “There is a thunderstorm coming. Is the house going to be OK?” Mary replies:*
Oh my, we have to go back — the windows are open!

Assuming that young children do not have the interpretive mechanisms available to simultaneously make sense of homogeneity and non-maximality, it might be reasonable for them to assign an existential interpretation to the definite plural in order to be able to accommodate such non-maximal uses. There is reportedly an asymmetry in the availability of non-maximal readings for affirmative and negated sentences, possibly related to the kinds of contexts in which we would use them (Križ, 2015b). If this is correct, then children will observe much fewer non-maximal readings of negated sentences, which could lead them to assume that such sentences are indeed only false when the predicate holds of none of the individuals in question. This, they can accommodate by assuming that the existentially interpreted definite plural has to take scope under negation^36^.

A second group of children was found to have already acquired scalar implicatures as well as a homogeneous interpretation of plural definite descriptions, and was therefore adult-like.

Finally, a third group of children appeared to access the homogeneous interpretation of the plural definite descriptions without computing scalar implicatures. A closer look in Experiment 2 suggests that some of these children actually assign a wide-scope universal interpretation to the definite plural. This would seem to be a natural hypothesis on the part of these children^37^, since, setting non-maximality aside, the data that are needed to distinguish this hypothesis from the correct homogeneous reading (e.g., involving definite plurals in the scope of non-monotonic quantifiers) are quite subtle and presumably not all too frequent in the speech children are exposed to. Importantly, however, there is still evidence for a group of children who do assign adult-like homogeneous readings to definite plurals while not computing scalar implicatures.

Given that (at least some) children start out with an existential meaning for definite plurals, and that by the time they have acquired scalar implicatures, they have also reached an adult-like homogeneous meaning for definite plurals, it is tempting to think that implicatures are, in fact, the way *by which* they obtain such a homogeneous meaning. This would accord exactly with Magri's (2014) implicature-based theory of homogeneity, in which definite plurals are assumed to have an existential literal meaning.

While it cannot be excluded that some children transition to the adult-like state via the implicature theory of homogeneity, our data provide evidence that the implicature theory is not a correct description of the adult state itself. Since the implicature theory requires the implicature from *some* to *not all* as a subcomputation of the implicature that is behind homogeneity effects, it predicts that homogeneous readings should not be more frequent than this scalar implicature. This is inconsistent with our adult data. If, however, as our data indicate, the implication between scalar implicatures and homogeneity is only unidirectional even in children (so that there are children with homogeneous definite plurals but no implicatures), it is also not clear that the implicature theory has a role to play in development. Rather, it seems quite plausible that the two phenomena are independent and that homogeneity (whatever its proper analysis) is simply acquired earlier than scalar implicatures^38^.

## 7. Conclusion

In this paper, we presented two experiments that tested children's interpretation of sentences containing plural definite descriptions, such as the affirmative *The trucks are blue* and the negated *The trucks are not blue*. These experiments also included testing children's ability to compute scalar implicatures, and therefore allowed us to directly compare children's performance on the two phenomena. This in turn afforded us the opportunity to assess the viability of scalar implicature accounts of homogeneity.

The data from our experiments confirm previous findings (Karmiloff-Smith, 1979; Caponigro et al., 2012) that (many) children interpret definite plurals as existential, and extend this existential interpretation to the context of negation, where we find that the existential takes low scope. This corresponds to the literal meaning hypothesized by the implicature theory of homogeneity (Magri, 2014). However, the finding of children (and adults) who have access to homogeneity while failing to compute the scalar implicature that is argued to be a sub-computation of homogeneity is incompatible with the predictions of this theory. While we have remained agnostic as to the nature of homogeneity in the adult grammar, our experiments suggest that it is a phenomenon distinct from scalar implicatures and acquired earlier by children.

## Data Availability Statement

The data and R scripts for this study are available online at: http://semanticsarchive.net/Archive/DM5YjA1M/Tieu-Kriz-Chemla-AcqHomogeneity.html.

## Ethics Statement

Ethical approval for this study was obtained from the CERES (Comité d'évaluation éthique des projets de recherche en santé non soumis à CPP) under approval number 2013/46.

## Author Contributions

LT, MK, and EC conceived and designed the study. LT prepared and carried out the experiments. MK performed the statistical analysis. All authors contributed to writing, revising, reading, and approving the submitted manuscript.

### Conflict of Interest

The authors declare that the research was conducted in the absence of any commercial or financial relationships that could be construed as a potential conflict of interest.

## Footnotes

^1^Note that homogeneity effects appear not only with definite plurals, but also when a predicate is ascribed to a single object that has parts to which the predicate is applicable: (ia) is true if the entire truck is blue, and (ib) is true if no appreciable part of the truck is blue.

- (i) a. The truck is blue.
- b. The truck isn't blue.

In this paper, however, we restrict our attention to the case of definite plurals.

^2^Here and elsewhere in the paper, we will sometimes sloppily use the term *definite description* to refer to a sentence containing a definite description in subject position.

^3^This pattern seems particularly plausible when one considers the kinds of contexts in which children might hear the plural definite being uttered. If indeed the positive (1) is only felicitous and true in contexts where all of the trucks are blue, the child should only ever hear such descriptions in scenarios that satisfy homogeneity. This could lead the child to form the generalization that plural definites have a universal meaning. Some empirical evidence for this state of affairs in the caregiver input may be found in corpus data reported by Caponigro et al. (2012). These authors examined child-directed speech in the CHILDES database (MacWhinney, 2000), and reported that all 6404 instances in their sample of plural definite descriptions headed by the determiner *the* referred to a maximal element. Note that evidence about negative plural definites may be more intricate, for reasons having to do with scope; if a child only ever hears the negative (2) in homogeneous contexts, the child might be led to liken the plural definite to a universal that must take wide scope with respect to negation.

^4^A reviewer points out that the current study might also be seen as a contribution to the broader investigation of the acquisition of predication, truth-value gaps, and negation more generally, that is, above and beyond definite plurals. In what follows, however, we will focus our attention specifically on how the child data can be brought to bear on the implicature approach to the phenomenon.

^5^We do not discuss here the very recent work by Bar-Lev (2018), which post-dates the writing of this paper. Bar-Lev proposes to view homogeneity as an implicature(-like) phenomenon in a way altogether different from Magri; the experimental data we present will not bear directly on Bar-Lev's approach.

^6^We have chosen to focus on the scalar implicature account primarily for practical reasons, as there is more existing work on the acquisition of implicatures than on the acquisition of vagueness or presupposition. We leave for future research an investigation of the predictions that alternative accounts of homogeneity may make for child language.

^7^Royle et al. (2018) report a replication of Munn et al.'s study in French, using the same act-out task to test (Canadian) French-speaking children's comprehension of requests such as: *Donne-moi les vaches qui sont à côté de la ferme* “Give me the cows that are beside the farm.” Children and adults generally gave maximal responses to definite plurals, with a significant effect of age observed for the children: the youngest children provided ~37% non-maximal responses while older children gave ~10% non-maximal responses (Royle et al., 2018:7).

^8^See Modyanova and Wexler (2008) for further evidence of non-maximal interpretations of free relatives.

^9^In fact, Caponigro et al.'s proposal would appear to suggest that young children don't initially fall into any of the three categories, or at least not systematically so.

^10^The reader might wonder whether Caponigro et al.'s (2012) *some* conditions could potentially speak to children's ability to compute scalar implicatures. The authors reported the percentage of maximal responses to *some*; notice that a maximal response would be consistent with the literal *some-or-all* meaning of the existential quantifier, but not with the *some-but-not-all* scalar implicature. Given this, it is striking to note that even 4- and 5-year-olds gave very few maximal responses in the *some* condition. This finding cannot be taken as conclusive evidence of calculation of the scalar implicature, however, for a couple of reasons. First, in an act-out task, participants may be driven to take the minimal action to satisfy a request; a less-than-maximal response, which involves less effort, would still be compatible with the literal interpretation of *some*. Second, in any given session, children were presented with requests containing *some* and requests containing *all*. Children could therefore have inferred that the use of *some* should elicit a different response from the use of *all*. Children have indeed been shown to be able to differentiate weak from strong scalar terms; such a finding, however, only establishes sensitivity to relative informativity, and may not necessarily signal the computation of implicatures (for relevant discussion, see Chierchia et al., 2001; Gualmini et al., 2001; Katsos and Bishop, 2011). Finally, we should make it very clear here that Caponigro et al. (2012) do not seek to make any claims about children's scalar implicatures. We simply point out here that although they included an existential quantification condition, we cannot draw on their results to make strong conclusions about children's performance on scalar implicatures.

^11^We varied the objects described and the colors of the objects in order to keep the task engaging for young children. We selected simple objects and colors that preschool-aged children would be familiar with, and ensured that pairs of colors were discernible for any colorblind participants. Children were also asked to verbally identify the objects (*Qu'est-ce que tu vois sur cette image?* “What do you see in this picture?”) and the colors of these objects. We did not systematically control for gender but aimed for a rough balance of masculine and feminine nouns across the experiment; of the six critical homogeneity targets, three were feminine (*les voitures, les étoiles, les balles*) and three were masculine (*les ballons, les camions, les coeurs*).

^12^Some native speakers of French may not find the negative (14b) to be an entirely natural formulation. We chose to place the negation before the universal quantifier for three reasons. First, a natural alternative would have been to float the universal quantifier, as in *Les coeurs ne sont pas tous rouges* “The hearts are not all red.” But we chose to avoid any potential issues related to children's mastery of floating quantification. Second, if we kept the universal quantifier in its unfloated position, as in *Tous les coeurs (ne) sont pas rouges* “All of the hearts are not red,” there was a question of whether children might be sensitive to the presence or absence of the *ne*. If, for whatever reason, participants failed to perceive the *ne*, for example, this could have invited an interpretation where the negation was phrasal rather than sentential, e.g., *Les coeurs sont [pas-rouges]* “The hearts are [not-red].” Finally, the version where *pas* precedes *tous* gives rise to the rather strong indirect scalar implicature that some of the hearts are red, which would serve to further emphasize the absence of homogeneity depicted in the test image.

^13^We chose to have three repetitions of each of the positive and negative homogeneity targets so as to keep the overall length of the experiment manageable for children; but a future study might ideally include more target trials, to ensure that a participant's categorization under this scheme truly reflects their interpretation of the definite description. In this respect, however, it is worth noting that our child participants were remarkably consistent in their responses to the targets, with only two of the children giving non-uniform responses (to the positive targets).

^14^Some of these justifications could be consistent with rejections not for non-homogeneity but rather for a failure to completely describe all of the objects in the picture. That is, the puppet only accurately described half of the objects. It is difficult to tease apart the two kinds of rejections here. Experiment 2 will include a control that allows us to address this potential concern.

^15^Unlike the child participants, no adult displayed the existential response pattern. Not all adults computed scalar implicatures, however. We will return to this point.

^16^The addition of varying intercepts and/or slopes in the models led to convergence problems and was therefore eschewed.

^17^Technically, the model had the form:

$$Y_{s,i}\sim bernoulli\left({logit}^{-1}\left(\pi_{s,i}\right)\right),\text{with}\pi_{s,i}=\alpha +\beta X_{\gamma \left(s\right)i}\text{and}X_{gi}\text{following}\text{Table 2}.$$

Bayesian models were fitted separately for children and adults using JAGS through the rjags package (Plummer, 2003). The prior for the intercept parameter α was set to a normal distribution with mean 0 and precision 0.001, while the prior for the slope parameter β was the non-negative half of the same distribution (since a participant cannot plausibly be more likely to judge a sentence true when it is, in fact, false). The prior for γ(*s*), the group parameter of each participant *s*, was uniform. 5,000 samples were drawn from each of 4 chains after 5,000 burn-in iterations.

^18^The group assignments remained the same when the model was fitted only on the items directly relevant to homogeneity and implicatures, i.e., the items in the the-gap-pos, the-gap-neg, and some-all-pos conditions.

^19^Deviating from Vehtari et al. (2017), we did not approximate the cross-validation by importance sampling, as it was questionable that our data set would meet the prerequisites for this procedure.

^20^$\underset{s}{\min}\left(\arg\underset{g}{\max}p\left(\gamma \left(s\right)=g|Y\right)\right)$, i.e., the minimal probability with which any subject was assigned its group was 0.82. The mean was 0.96.

^21^At least not regardless of context, and participants may differ in what kind of context they assume to obtain in the experimental situation.

^22^The relevant evidence comes from definite plurals embedded in non-monotonic contexts, as well as definite plurals with a pronoun bound by a higher negative quantifier (cf. Magri, 2014 and Križ and Chemla, 2015).

^23^This is equivalent to interpreting negation as weak negation (~*p* is true if *p* is not true) instead of strong negation (¬*p* is true if *p* is false) in a trivalent logic.

^24^The nature of the seemingly universal responses from some adults is irrelevant to this argument.

^25^For adult participants, half of the controls involved the all context and half involved the none context.

^26^This control also allowed us to ensure that the children could access adult-like interpretations of existentially quantified sentences.

^27^We provide the “not all” translation here, as it more accurately reflects the most natural interpretation of the French sentence, i.e., with negation scoping over the universal. The “all not” translation is ambiguous in English.

^28^Unlike the negative universal sentences in Experiment 1, the universal quantifier here preceded the negation. Since we intended to use this condition as a scope ambiguity control for the homogeneity targets, we wanted to ensure that the sentences in the two conditions were as parallel as possible, in particular with respect to the relative surface order of the quantifier and negation.

^29^It is worth noting that children were target-like on the same controls in Experiment 1. It's not entirely clear why the ternary task should bring out the infelicity of the negative descriptions more than the binary task. One possible reason is that choosing from three response options instead of two was more demanding, which could have pushed children to rely on a superficial strategy of quickly rejecting (or “punishing” the puppet for) any sentences that they perceived to be irrelevant. For example, they could have checked whether any of the objects in the picture matched the color mentioned by the puppet; if not, the puppet's statement would quickly be rejected as irrelevant or infelicitous.

^30^Again, some would prefer an analysis that does not bin participants into categories, but we include this discussion here to explore the possible interpretive preferences observable in our two participant groups; as in Experiment 1, participants turned out to align remarkably well into a subset of the possible categories.

^31^The models were thus similar to the ones used in the previous experiment, except that we performed ordinal regression because the responses could now take 3, and not only 2, values. The analysis was again performed with JAGS/rjags (Plummer, 2003). The prior for the two threshold parameters in the ordinal regression was set to a normal distribution with mean 0 and precision 0.001, while the prior for the slope parameter was set to the positive half of the same distribution. The prior over the group parameter γ was uniform (over those groups which were available to the model). Leave-one-out cross-validation was performed as before on the basis of 5,000 samples after 5,000 burn-in iterations from 4 chains, for a total of 20,000 samples per model and data point.

^32^Since hom and pt do not differ on the conditions under consideration (but only on all-gap-pos, which was not included), the latter group was excluded from the models. Note that while we did take into account the possibility of scope ambiguity effects, we did not use the all-gap-neg data points to estimate their prevalence. The effect of the scope ambiguity of the negative universal sentences was very strong in that all possible responses were chosen in a sizeable percentage of cases. Given that this is not so for negative definite description sentences, these effects are clearly more prevalent with the universal *all*, and we found that the scope ambiguity effect with *all* dominated the group assignment choices of the models at the expense of an accurate categorization on the basis of the definite description data. In other words, the model would rather give up accuracy on the definite description data than miss the scope ambiguity effects with *all*. Furthermore, there was no a priori reason to expect the scopal behavior of different quantifiers (in this case, definite descriptions and *all*) to be the same. We thus concluded that the inclusion of these data points would hamper, rather than improve, the analysis.

^33^The top model also included the ws/−si group. It should, however, be noted that the models are likely to overestimate the prevalence of the ws/−si group. Both our own adult data and the data from Križ and Chemla (2015) suggest that adults often judge sentences as false when they are really undefined due to a homogeneity violation. Adults do this more often than they judge the same sentences true, and also more often than they judge a sentence with a false implicature as false (cf. Figures 12, 13 above). Our simple ordinal regression models do not account for this fact and therefore categorize children into the ws/−si group even when they are really in the hom/−si group, simply translating the underlying undefined status of the sentence to a minimal response.

^34^The only exception was sa/−si, which was independently the worst model. The reason why sa/−si is a bit better in Analysis 2 is presumably that it now has a way of assigning children to a ws/−si group by sacrificing fit on the implicature data: (some) children that ought to be in ws/−si were instead categorized as ws/+si. This is not possible in Analysis 1, where no ws/+si group exists.

^35^If we look at how the maximal model categorizes children (by maximal posterior probability of group), we find that only one child is assigned to sa/+si and no child is assigned to ws/+si, whereas there are six participants in the hom/+si group. In light of this model's failure to perform markedly better than more parsimonious ones, we conclude that it is probably overfitted and that the assumption that implicatures imply homogeneity need not be given up on the basis of these results.

^36^Note that a low-scope universal reading for definite plurals is, in light of this input, an implausible hypothesis, such that its absence in children is not surprising.

^37^Indeed, Caponigro et al.'s (2012) sample of plural definite descriptions in child-directed speech is entirely associated with maximal interpretations.

^38^The ages of the groups in Experiment 1 show a trend toward children without scalar implicatures being younger than those with scalar implicatures, but the difference is not statistically significant in our sample, which was drawn from the rather narrow range of 4;04, 14−5;03, 24. The main argument is, of course, the unidirectional implication from scalar implicatures to homogeneity that we seem to observe.
