# Supplementary material for: Children's Acquisition of Homogeneity in Plural Definite Descriptions
Source: Front Psychol. 2019 Nov 6;10:2329. doi: 10.3389/fpsyg.2019.02329 (PMC6856947; doi:10.3389/fpsyg.2019.02329)
Supplement: Supplementary file 1 [file Data_Sheet_1.pdf]

Appendix

A Test sentences and contexts

A.1 Experiment 1

(1) Training items

a.	La chaise est rose.	‘The chair is pink.’
b.	Le piano est orange.	‘The piano is orange.’

(2) Positive sentences containing plural definite descriptions, presented in GAP contexts (positive homogeneity targets)

a.	Les ballons sont rouges.	‘The balloons are red.’
b.	Les voitures sont bleues.	‘The cars are blue.’
c.	Les étoiles sont jaunes.	‘The stars are yellow.’

(3) Negative sentences containing plural definite descriptions, presented in GAP contexts (negative homogeneity targets)

a.	Les balles ne sont pas rouges.	‘The balls are not red.’
b.	Les camions ne sont pas bleus.	‘The trucks are not blue’.
c.	Les coeurs ne sont pas jaunes.	‘The hearts are not yellow.’

(4) Positive sentences containing plural definite descriptions, in ALL/NONE contexts (unambiguous true/false controls)

a.	Les parapluies sont rouges.	‘The umbrellas are red.’
b.	Les bateaux sont bleus.	‘The boats are blue.’
c.	Les crayons sont jaunes.	‘The crayons are yellow.’
d.	Les feuilles sont vertes.	‘The leaves are green.’

(5) Negative sentences containing plural definite descriptions, in NONE/ALL contexts (unambiguous true/false controls)

a.	Les bottes ne sont pas rouges.	‘The boots are not red.’
b.	Les trains ne sont pas bleus.	‘The trains are not blue.’
c.	Les pinceaux ne sont pas jaunes.	‘The paintbrushes are not yellow.’
d.	Les chaussures ne sont pas roses.	‘The shoes are not pink.’

(6) Existentially quantified sentences, presented in ALL contexts
(scalar implicature targets)

a.	Certains chapeaux sont roses.	‘Some hats are pink.’
b.	Certaines fleurs sont vertes.	‘Some flowers are green.’
c.	Certaines tentes sont oranges.	‘Some tents are orange.’
d.	Certains sacs sont bleus.	‘Some bags are blue.’

(7) Positive universally quantified sentences, presented in GAP contexts
(positive universal controls)

a.	Toutes les tasses sont roses.	‘All the cups are pink.’
b.	Toutes les boîtes sont vertes.	‘All the boxes are green.’

c. Tous les pulls sont oranges. ‘All the sweaters are orange.’

(8) Negative universally quantified sentences, presented in GAP contexts (negative universal controls)

a. Pas tous les bols sont roses. ‘Not all the bowls are pink.’
b. Pas toutes les maisons sont vertes. ‘Not all the houses are green.’
c. Pas tous les gants sont oranges. ‘Not all the gloves are orange.’

A.2 Experiment 2

(9) Training items

a. La chaise est rose. ‘The chair is pink.’
b. Le piano est orange. ‘The piano is orange.’

(10) Positive sentences containing plural definite descriptions, presented in GAP contexts (positive homogeneity targets)

a. Les ballons sont rouges. ‘The balloons are red.’
b. Les voitures sont bleues. ‘The cars are blue.’
c. Les étoiles sont jaunes. ‘The stars are yellow.’

(11) Negative sentences containing plural definite descriptions, presented in GAP contexts (negative homogeneity targets)

a. Les balles ne sont pas rouges. ‘The balls are not red.’
b. Les camions ne sont pas bleus. ‘The trucks are not blue.’
c. Les coeurs ne sont pas jaunes. ‘The hearts are not yellow.’

(12) Positive sentences containing plural definite descriptions, in ALL/NONE contexts (unambiguous true/false controls)

a. Les parapluies sont rouges. ‘The umbrellas are red.’
b. Les bateaux sont bleus. ‘The boats are blue.’
c. Les crayons sont jaunes. ‘The crayons are yellow.’
d. Les feuilles sont vertes. ‘The leaves are green.’

(13) Negative sentences containing plural definite descriptions, in NONE/ALL contexts (unambiguous true/false controls)

a. Les bottes ne sont pas rouges. ‘The boots are not red.’
b. Les trains ne sont pas bleus. ‘The trains are not blue.’
c. Les pinceaux ne sont pas jaunes. ‘The paintbrushes are not yellow.’
d. Les chaussures ne sont pas roses. ‘The shoes are not pink.’

(14) Existentially quantified sentences, presented in ALL contexts (scalar implicature targets)

a. Certains chapeaux sont roses. ‘Some hats are pink.’
b. Certaines tentes sont oranges. ‘Some tents are orange.’
c. Certains sacs sont bleus. ‘Some bags are blue.’

(15) Existentially quantified sentences, presented in GAP contexts (incomplete description controls)

a.	Certains parapluies sont rouges.	‘Some umbrellas are red.’
b.	Certains crayons sont jaunes.	‘Some crayons are yellow.’
c.	Certaines bottes sont rouges.	‘Some boots are red.’

(16) Positive universally quantified sentences, presented in GAP contexts (partial truth controls)

a.	Toutes les tasses sont roses.	‘All the cups are pink.’
b.	Toutes les boîtes sont vertes.	‘All the boxes are green.’
c.	Tous les pulls sont oranges.	‘All the sweaters are orange.’

(17) Negative universally quantified sentences, presented in GAP contexts (scope ambiguity controls)

a.	Pas tous les bols sont roses.	‘Not all the bowls are pink.’
b.	Pas toutes les maisons sont vertes.	‘Not all the houses are green.’
c.	Pas tous les gants sont oranges.	‘Not all the gloves are orange.’

B Instructions for ternary judgment task (Exp. 2)

B.1 French

“Boba adore écouter des histoires, mais parfois il ne fait pas très attention. Toi, tu vas à l’école, tu as déjà quatre ans et demi, tu sais faire attention ! Mais Boba est trop petit ! Alors ce qu’on va faire c’est qu’on va regarder des images avec plein de choses intéressantes et après on va demander à Boba de nous dire quelque chose sur les images. Et toi tu vas décider si Boba a bien répondu ou pas. Je vais te montrer comment faire. Tu vois les fraises ? C’est son fruit préféré ! Boba adore les fraises ! Si Boba répond parfaitement, tu peux lui donner trois fraises ! Tu prends ça et tu mets dans la boîte, comme ça ! Il va être très content, parce qu’il adore les fraises ! Si Boba dit une bêtise, s’il ne fait pas attention, s’il ne répond pas bien du tout, tu vas lui donner une seule fraise ! Tu vois, tu prends et tu mets dans la boîte ! Et si c’est pas parfait, mais c’est pas trop mauvais, c’est moyen, c’est un peu entre les deux, tu peux lui donner deux fraises ! Tu vois ? OK, on va répéter ! Si Boba répond parfaitement, si c’est très bien, tu lui donnes combien de fraises ? [...] Et si Boba dit une bêtise tu lui donnes combien de fraises ? [...] Et si c’est moyen, ce n’est ni parfait ni mauvais, tu lui donnes combien de fraises ? [...] Parfait ! C’est parti !”

B.2 English

“Boba loves to listen to stories, but sometimes he doesn’t pay very much attention. You go to school, you’re already four and a half, you know how to pay attention! But Boba is too little! So what we’re going to do is look at some pictures with lots of interesting things and then we’re going to ask Boba to tell us something about the pictures. And you’re going to decide if Boba answered well or not. I’ll show you how. You see the strawberries? They’re his favourite fruit! Boba loves strawberries! If Boba answers perfectly, you can give him

three strawberries! You take this *[card with three strawberries]* and you put it in the box, like this! He's going to be very happy, because he loves strawberries! If Boba says something silly, if he doesn't pay attention, if he doesn't answer well at all, you're only going to give him one strawberry! You see, you take it and you put it in the box! And if it's not perfect, but not too bad, if it's average, a bit in between the two, you can give him two strawberries! You see? OK, let's go through it one more time. If Boba answers perfectly, if it's very good, you give him how many strawberries? [...] And if Boba says something silly, you give him how many strawberries? [...] And if it's average, it's not perfect or bad, you give him how many strawberries? [...] Perfect! Let's start!"
